# Sex differences in the polygenic architecture of hearing problems in adults

**DOI:** 10.1186/s13073-023-01186-3

**Published:** 2023-05-11

**Authors:** Flavio De Angelis, Oana A. Zeleznik, Frank R. Wendt, Gita A. Pathak, Daniel S. Tylee, Antonella De Lillo, Dora Koller, Brenda Cabrera-Mendoza, Royce E. Clifford, Adam X. Maihofer, Caroline M. Nievergelt, Gary C. Curhan, Sharon G. Curhan, Renato Polimanti

**Affiliations:** 1 Department of Psychiatry, Yale University School of Medicine, 60 Temple, Suite 7A, New Haven, CT USA; 2 Veteran Affairs Connecticut Healthcare System, West Haven, CT USA; 3 Channing Division of Network Medicine, Department of Medicine, Brigham and Women’s Hospital, Boston, MA USA; 4 Harvard Medical School, Boston, MA USA; 5 Department of Biology, University of Rome “Tor Vergata”, Rome, Italy; 6 Department of Genetics, Microbiology and Statistics, Faculty of Biology, University of Barcelona, Barcelona, Spain; 7 Division of Otolaryngology, Department of Surgery, University of California, San Diego, La Jolla, CA USA; 8 Research Service, Veterans Affairs San Diego Healthcare System, San Diego, CA USA; 9 Department of Psychiatry, University of California, San Diego, La Jolla, CA USA; 10 Center of Excellence for Stress and Mental Health, Veterans Affairs San Diego Healthcare System, San Diego, CA USA

**Keywords:** Hearing problems, Genome-wide association study, Ancestry; Polygenic risk scores, Transcriptomic regulation, Sex differences, Pleiotropy, Causal inference, Genome-wide gene-by-environment interaction

## Abstract

**Background:**

Hearing problems (HP) in adults are common and are associated with several comorbid conditions. Its prevalence increases with age, reflecting the cumulative effect of environmental factors and genetic predisposition. Although several risk loci have been already identified, HP biology and epidemiology are still insufficiently investigated by large-scale genetic studies.

**Methods:**

Leveraging the UK Biobank, the Nurses’ Health Studies (I and II), the Health Professionals Follow-up Study, and the Million Veteran Program, we conducted a comprehensive genome-wide investigation of HP in 748,668 adult participants (discovery *N* = 501,825; replication *N* = 226,043; cross-ancestry replication *N* = 20,800). We leveraged the GWAS findings to characterize HP polygenic architecture, exploring sex differences, polygenic risk across ancestries, tissue-specific transcriptomic regulation, cause-effect relationships with genetically correlated traits, and gene interactions with HP environmental risk factors.

**Results:**

We identified 54 risk loci and demonstrated that HP polygenic risk is shared across ancestry groups. Our transcriptomic regulation analysis highlighted the potential role of the central nervous system in HP pathogenesis. The sex-stratified analyses showed several additional associations related to peripheral hormonally regulated tissues reflecting a potential role of estrogen in hearing function. This evidence was supported by the multivariate interaction analysis that showed how genes involved in brain development interact with sex, noise pollution, and tobacco smoking in relation to their HP associations. Additionally, the genetically informed causal inference analysis showed that HP is linked to many physical and mental health outcomes.

**Conclusions:**

The results provide many novel insights into the biology and epidemiology of HP in adults. Our sex-specific analyses and transcriptomic associations highlighted molecular pathways that may be targeted for drug development or repurposing. Additionally, the potential causal relationships identified may support novel preventive screening programs to identify individuals at risk.

**Supplementary Information:**

The online version contains supplementary material available at 10.1186/s13073-023-01186-3.

## Background

Acquired hearing problems (HP) are the third most common chronic health condition [1] and the fourth leading cause of disability globally [2]. The World Health Organization (WHO) reports that nearly 2.5 billion individuals will have some degree of HP by 2050 [3]. HP in adults is associated with several comorbid conditions [4]. For instance, HP-induced impaired communication ability particularly among older people can lead to social isolation with major health, psychosocial, and economic consequences, reducing the quality of life [4]. HP affects individuals of all ages, but its prevalence increases with age, reflecting the cumulative effect of environmental factors and genetic predisposition [5].

Heritability estimates from family studies of HP range from 30 up to 70% [6], highlighting that genetic variation is a key determinant for individual HP risk. More than 100 genes present mutations that result in congenital HP not associated with disorders in other organs or dysmorphic features (non-syndromic HP) [7]. Mutations causing congenital HP affect genes involved in cochlear function, specifically affecting the sensory and mechanosensory cells [8]. Beyond these Mendelian forms, acquired HP appears to be due to the additive contribution of many common genetic variants with small individual effects. Large-scale genome-wide association studies (GWAS) conducted in population-based cohorts have identified more than 50 common risk variants and characterized the regulatory role of these loci in multiple cell and tissue types [9–11]. Although these studies have generated important insights into the genetic predisposition to HP in adults, several aspects of HP pathogenesis are still unclear. For instance, HP among older adults is more common, more severe, and with earlier onset in men than in women, even after adjusting for confounding factors such as higher occupational noise exposure in men [12]. However, the molecular pathways that underlie HP sex differences are unclear. Similarly, we have a limited understanding of the biological processes interacting with HP environmental risk factors. Several studies showed that noise pollution and tobacco smoking are HP risk factors [13–15], but to date no large-scale studies investigated how genetic variation interacts with noise pollution and tobacco smoking in determining HP risk.

In the present study, we conducted an extensive genome-wide investigation across the UK Biobank (UKB; 251,233 women and 214,549 men) [16], the Nurses’ Health Studies (NHS I, 14,978 women; NHS II, 12,533 women) [17] and the Health Professionals Follow-up Study (HPFS, 8532 men) [18]. The risk loci identified were replicated in a sample of 226,043 participants (93% males) from the Million Veteran Program (MVP) [19]. Our findings provide a more comprehensive understanding of the genetic basis of HP sex differences, uncovering novel sex-specific risk loci, molecular processes, putative cause-effect relationships, and the interaction of genetic variation with sex, noise pollution, and smoking behaviors.

## Methods

### Cohorts and hearing-problem assessment

Leveraging genome-wide information from UKB, NHS I, NHS II, HPFS, and MVP cohorts, we investigated the polygenic architecture of HP in 748,668 adult participants (discovery *N* = 501,825; replication *N* = 226,043; cross-ancestry replication *N* = 20,800).

UKB is a large population-based research resource, containing in-depth genetic and health information from over 500,000 UK participants 40–69 years at enrollment [16]. UKB HP-related phenotypes were defined by self-reported items derived from a touchscreen questionnaire and audiometric measurements assessed through the Speech Recognition Threshold (SRT) test. The self-reported items included the following binary traits: “Do you have any difficulty with your hearing?” (UKB Field ID: 2247); “Do you find it difficult to follow a conversation if there is background noise (such as TV, radio, children playing)?” (UKB Field ID: 2257); and “Do you use a hearing aid most of the time?” (UKB Field ID: 3393). UKB participants who indicated they were completely deaf (*N* = 144) were excluded from the analysis to reduce the likelihood of including congenital forms of HP [11]. After excluding deaf individuals, the number of individuals with less severe congenital HP are expected to be negligible, because of the low prevalence of congenital HP. For ~ 12% of the UKB participants (59,807 for UKB Field ID 2247; 60,448 for UKB Field ID 2257; and 40,656 for UKB Field ID 3393), these items were assessed multiple times. Since age is a strong risk factor for acquired HP, we considered the most recent assessment when multiple assessments were available to improve the ability to detect the disease onset. In addition to considering these binary traits individually, we combined them in a four-category ordinal phenotype (Additional file 1: Table S1). For the SRT-derived audiometric measurements, we considered the UKB item “the signal-to-noise ratio at which half of the presented speech can be understood correctly” for both left and right ears (UKB Field ID: 20,019 and 20,021, respectively). Because of the much larger sample size, we included unrelated UKB participants of European descent (EUR) in the primary discovery sample. The other ancestry groups available in UKB were used for single-variant and polygenic risk score (PRS) replication (see section “Cross-ancestry replication and polygenic risk scoring”).

Additional genome-wide information was derived from NHS I, NHS II, and HPFS cohorts. The NHS I began in 1976 when 121,700 female registered nurses, aged 30–55 years, were enrolled by completing a baseline questionnaire about their health and lifestyle [20]. In 1989, the NHS II was established and enrolled 116,429 younger female registered nurses, aged 25 to 42 years. The HPFS began in 1986 and enrolled 51,529 male health professionals, aged 40 to 75 years [18]. In each of the cohorts, detailed information on demographics, health, diet, and lifestyle factors was collected and updated every 2 years (every 4 years for diet). The follow-up rates in all 3 cohorts exceed 90% of eligible person-time [20]. Self-reported hearing status was determined based on participants’ responses to biennial questionnaires. “Hearing difficulty” was defined as a participant report of a hearing problem that was mild, moderate, severe (non-hearing aid user), or severe (hearing aid user). Leveraging samples with genome-wide information, we investigated 14,978 unrelated female NHS I participants (56% cases), 12,533 unrelated female NHS II participants (36% cases), and 8,532 unrelated male HPFS participants (60% cases), all of EUR descent. To maximize the sample size available, we meta-analyzed NHS I and II cohorts and referred to them hereafter as a single NHS sample.

The MVP is a biobank funded by the US Department of Veterans Affairs that to date enrolled more than 800,000 participants among active users of the Veterans Health Administration healthcare system [19]. In our analysis, we used genome-wide information regarding 85,743 cases and 140,300 controls (93% males) based on self-reported hearing problems. Information regarding HP assessment in MVP has been previously described [21]. Briefly, cases were defined as (i) those that reported HP in the MVP baseline questionnaire or (ii) those with a diagnosis of sensorineural HP in the electronic health record (EHR), but not mixed hearing loss, conductive hearing loss, ototoxic hearing loss, or sudden hearing loss.

### Genome-wide data quality control and GWAS meta-analysis

In the present study, we analyzed multiple samples that were genotyped with different arrays. Quality control (QC) of the genetic data was conducted in accordance with cohort-specific criteria to ensure that high-quality data were used in the discovery meta-analysis of the UKB, NHS, and HPFS samples and in the subsequent replication conducted in the MVP sample. Accordingly, some of the QC cutoffs applied varied across the cohorts investigated.

Our primary analysis was conducted in UKB EUR participants. Considering this ancestry group, we used UKB data imputed using the Haplotype Reference Consortium [16], filtering variants with imputation INFO score < 0.8, minor allele frequency (MAF) < 0.01, Hardy–Weinberg equilibrium (HWE) *p*-values < 10^−6^, and missingness > 0.1. After quality control, we analyzed up to 9,547,865 variants. For ancestry, sex, and relatedness assignments, we used definitions from the Pan-UKB analysis [22]. Briefly, related individuals were removed considering a kinship coefficient > 0.042. UKB participants with sex chromosome aneuploidies were excluded from the analysis. Ancestry was assigned using a random forest classifier based on features predictive of similarities with reference populations derived from a combined 1000 Genomes Project plus Human Genome Diversity Panel. This is in line with the recent report of the US National Academies of Sciences, Engineering, and Medicine [23] recommending that population descriptors in genetic research should be based on genetic similarities with reference populations rather than ethnicity-based classification. Additionally, Pan-UKB ancestry assignments permitted us to retain a large number of UKB participants in our study [22]. To account for population structure within European populations, we used within-ancestry principal components (PC) previously calculated in the Pan-UKB analysis.

In the NHS and HPFS cohorts, quality checks included the exclusion of those participants with a poor genotype call rate (< 95%) and a check for relatedness. A detailed description of quality control criteria was previously reported [24]. Briefly, a pairwise identity-by-descent analysis to estimate relatedness among NHS and HPFS participants. As also previously described [24], ancestry assignment and population structure covariates were derived from cross-ancestry and within-ancestry PC analyses, respectively. SNPs with a poor call rate (< 95%), out of HWE (*P* < 10^−5^), with high duplicate discordance rates, or that are monomorphic were excluded. Genotyped data from each of the NHS and HPFS studies were imputed using the 1000 Genomes Project Phase 3 reference panel. After imputation, SNPs with imputation INFO score < 0.8 or MAF < 0.05 were excluded. We analyzed a total of 5,550,954 and 5,549,467 imputed variants in HPFS and NHS samples, respectively.

Details regarding genotyping and imputation of the MVP samples were described elsewhere [25]. Relatedness among MVP participants was estimated using KING [26]. For each pair of subjects with an estimated kinship coefficient > 0.088 (2nd degree or closer), one individual was removed, with the preference to retain cases. If individuals had the same diagnostic status, one individual was removed at random. HARE (harmonized ancestry and race/ethnicity) estimates [27] were used to select European-ancestry individuals.

GWAS were carried out in the UKB, NHS, and HPFS by logistic regression (case–control HP phenotype coded as dependent variable), using PLINK 2.0 [28] and including age and the top-10 within-ancestry PCs as covariates. For the sex-combined analyses, sex was also included as a covariate. The GWAS generated by the individual cohorts were meta-analyzed using the inverse variance-based method implemented in METAL [29], using SCHEME STDERR option. Genetic associations were considered significant when surviving the genome-wide multi-testing correction (*p* < 5 × 10^−8^). The number of variants shared across the three cohorts was 5,331,308.

We defined sex-specific genetic associations comparing sex-specific effect sizes among those surviving genome-wide multiple testing correction in one of the two sexes (*p* < 5 × 10^−8^). Statistical differences between sex association statistics were calculated using *z*-test: $z=({\mathrm{beta}}_{\mathrm{female}}-{\mathrm{beta}}_{\mathrm{male}})/\sqrt{{\mathrm{SE}}_{\mathrm{female}}^{2}+{\mathrm{SE}}_{\mathrm{male}}^{2}}$. *Z* scores were then converted to two-tail *p*-values, hereafter referred as difference-*p*.

In MVP, the replication of the loci identified in the UKB-NHS-HPFS GWAS meta-analysis was performed using logistic regression of the HP phenotype (case–control variable coded as the dependent term) on imputed SNP dosages including as covariates: the top-10 within-ancestry PCs, age, and sex, using PLINK 1.9 software [28]. In addition to performing a single-variant replication, we also tested the association of a PRS derived from the UKB-NHS-HPFS GWAS meta-analysis with HP assessed in the MVP cohort. The analysis was conducted with PRSice v. 2.3.1.c [30], using the clumping-thresholding method to maximize the predictive ability of the derived polygenic scores [31]. Using 1000 Genomes Project EUR populations as reference panel, SNPs were clumped based on 250-kb windows, based on clump-r2 threshold = 0.1 and clump-p threshold = 1, respectively. The step size of the threshold was set to 5 × 10^−5^, and the range of *p*-value thresholds was from 5 × 10^−8^ to *p* = 1 using an additive model for regression at each threshold. We included as covariates the top-10 within-ancestry principal components, age, and sex. For each threshold, PRS R^2^ statistics (i.e., phenotypic variance explained by the PRS) is calculated as: $1 - ( 1 - {R}_{\mathrm{FULL}}^{2}) / ( 1 - {R}_{\mathrm{NULL}}^{2})$, where ${R}_{\mathrm{FULL}}^{2}$ is the phenotypic variance explained by the full model and ${R}_{\mathrm{NULL}}^{2}$ is the phenotypic variance explained by the model including only the covariates.

### SNP-based heritability and genetic correlation

SNP-based heritability (SNP-h^2^) and genetic correlation (rg) for all hearing traits were estimated using the linkage disequilibrium score regression (LDSC) method [32]. The analysis was conducted considering the HapMap 3 reference panel and pre-computed LD scores based on the 1000 Genomes Project reference data for EUR individuals. SNP-h^2^ and genetic correlations were evaluated among HP traits assessed in the UKB, NHS, HPFS, and their meta-analyses. Since functional categories of the genome contribute disproportionately to the heritability of complex diseases, SNP-h^2^ partitioning was conducted with LDSC using 95 baseline genomic annotations such as allele frequency distributions, conserved genomic regions, regulatory elements, and annotations for genic, loss-of-function (LoF) intolerant, and positively and negatively selected regions [33]. Bonferroni correction accounting for the number of annotations tested (*p* < 5.26 × 10^−4^) was applied to define significant SNP-h^2^ enrichments.

We also used the LDSC method [32] to conduct a phenome-wide genetic correlation analysis of HP, testing 7153 phenotypes for the sex-combined investigation, 3287 phenotypes for the female-specific investigation, and 3144 phenotypes for the male-specific investigation. Bonferroni correction accounting for the number of phenotypes tested was applied to define significant genetic correlations in the sex-combined and sex-stratified phenome-wide analyses (sex-combined *p* < 6.99 × 10^−6^; female-specific *p* < 1.52 × 10^−5^; male-specific *p* < 1.59 × 10^−5^). To maximize the statistical power of this analysis, we used European-ancestry genome-wide association statistics for the sex-combined analysis available in the Pan-UKB data release [22]. We used Pan-UKB genome-wide association statistics generated from other ancestry groups in a cross-ancestry analysis described below (see “Cross-ancestry replication and polygenic risk scoring”). Sex-specific genome-wide association statistics generated from European-descent participants were derived from a previous UKB analysis [34].

### Latent causal variable analysis

To evaluate whether the genetic correlations of HP are due to cause-effect relationships, we used the latent causal variable (LCV) method to conduct a genetically informed causal inference analysis [35]. As recommended, only SNPs with MAF > 5% were considered, and the major histocompatibility region was removed. Considering traits that reached at least a nominally significant genetic correlation (*p* < 0.05) with HP, we tested 879 traits in the sex-combined analysis, 323 traits in the female-specific analysis, and 332 traits in the male-specific analysis. For each comparison, the genetic causality proportion (gcp) can range from zero (no partial genetic causality) to one (full genetic causality). Positive and negative gcp values reflect the direction of the putative causal effect (i.e., phenotype #1 → phenotype #2 and phenotype #2 → phenotype #1, respectively). Information regarding the sign of the LCV effect is provided by the LCV rho statistics: rho > 0 corresponds to a positive effect while rho < 0 corresponds to a negative effect. To define statistically significant gcp estimates, we applied a multiple testing correction accounting for the number of phenotypes tested (sex-combined *p* < 5.69 × 10^−5^; female-specific *p* = 1.55 × 10^−4^; male-specific *p* < 1.51 × 10^−4^).

### Variant prioritization, fine-mapping, and multi-tissue transcriptome-wide association study

To identify the causal loci underlying the statistical association observed, we functionally annotated GWAS findings and prioritized the most likely causal SNPs and genes using pre-calculated LD structure based on 1000 Genomes Project EUR reference populations. The risk loci identified in the sex-combined and sex-specific meta-analyses were classified considering the following parameters derived from Functional Mapping and Annotation of Genome-Wide Association Studies (FUMA) [36]: leadP = 5 × 10^−8^; gwasP = 0.05; R^2^ = 0.1; refpanel = 1000 Genomes Project Phase3 EUR reference populations; MAF = 0.01; refSNPs = 1; mergeDist = 250. Positional mapping was performed considering MapWindowSize = 10 and the minimum Combined Annotation Dependent Depletion (CADD) [37] score for SNP filtering was set to 0. Gene-based analysis and gene-set analysis were performed with Multi-marker Analysis of GenoMic Annotation (MAGMA) [38] integrated into FUMA. Tissue enrichment analysis was performed using Genotype-Tissue Expression (GTEx) project v8 data [39].

We fine-mapped the association statistics for a 3-Mb region around the lead SNP of the genomic risk locus identified by FUMA (r^2^ = 0.1; window = 250 bp). Each region for the respective association was fine-mapped to determine 95% credible set (i.e., sets of putative causal variants within GWAS-identified loci) using susieR [40] with at most 10 causal variants (default). The credible set reports variants most likely to be causal based on the marginal posterior inclusion probability (PIP) ranging from 0 to 1, with values closer to 1 to be most causal.

To further explore transcriptomic regulation in the context of HP, we performed a multi-tissue TWAS (transcriptome-wide association study), using the S-MultiXcan approach to combine information across 49 GTEx tissues adjusting for tissue–tissue correlation [41]. Significant transcriptomic associations were defined as those surviving Bonferroni correction accounting for the number of genes tested (*N* = 22,335; *p* < 2.24 × 10^−6^).

### Cross-ancestry replication and polygenic risk scoring

We used Pan-UKB data [22] to conduct cross-ancestry replication and PRS analyses. We decided to not include these data in the discovery meta-analysis, because they would provide only a 4% increase in the discovery sample size. We derived a PRS from the UKB-NHS-HPFS GWAS meta-analysis that was tested in UKB participants of African (AFR *N* = 6636, 11% cases), admixed American (AMR *N* = 980, 16% cases), Central/South Asian (CSA *N* = 8876, 17% cases), East Asian (EAS *N* = 2709, 12% cases), and Middle Eastern (MID *N* = 1599, 16% cases) ancestries (target datasets). A total of 9,781,251 variants were analyzed in the Pan-UKB analysis. In addition to performing a single-variant replication, we also conducted a cross-ancestry PRS analysis. This was performed by analyzing genome-wide association statistics (UKB-NHS-HPFS GWAS meta-analysis as base and Pan-UKB GWAS as target) using the gtx R package incorporated in PRSice v1.25 [42]. Although this approach is different from the one used in the PRS analysis in the MVP, they are both based on clumping-thresholding and the results obtained are comparable. Both sets of genome-wide association statistics were generated including age, sex, and within-ancestry principal components as covariates. An approximate estimate of the explained variance (i.e., R^2^ statistics) was calculated from a multivariate regression model [43]. The PRSs were calculated after using *P*-value–informed clumping with an LD cutoff of R^2^ = 0.1 within a 250-kb window. Because the training dataset (i.e., UKB-NHS-HPFS GWAS) was generated from EUR individuals, we used EUR samples from the 1000 Genomes Project as the LD reference panel. False discovery rate (FDR) accounting for the number of *p*-value thresholds tested was considered to define PRS associations surviving multiple testing correction.

### Multivariate gene-by-environment genome-wide interaction study

To explore further the interplay of HP genetics with non-genetic factors beyond sex differences (investigated in our sex-specific GWAS), we conducted a multivariate gene-by-environment genome-wide interaction study (GEWIS) using StructLMM [44]. This is a linear mixed model approach to efficiently detect interactions between loci and multiple potentially correlated environments [44]. Since StructLMM assumes a quantitative trait, the GEWIS was conducted considering the four-category ordinal trait described above (Additional file 1: Table S1). Because of the known association of noise pollution and tobacco smoking with HP [13–15], the multivariate GEWIS was performed considering sex, smoking behaviors and exposures, and different types of noise pollution exposure (Additional file 1: Table S2). Due to the limited depth of information in other cohorts and to maximize statistical power, the analysis was limited to UKB and to items assessed in the majority of UKB participants. Age, age^2^, and top-10 within-ancestry PCs were included as covariates. Since the environmental factors entered are converted to a covariance matrix by the StructLMM approach [44], no issue (e.g., increased multiple testing) or bias (e.g., collinearity) is expected due to the degree of correlation among the environmental factors investigated. Marginal log-likelihoods (log (Bayes factor -BF)) between the full model and the reduced model with environments removed were used to identify the most relevant environments for the detected locus interaction effects. A pathway enrichment analysis was conducted considering loci with nominally significant multivariate gene-environment interaction. These variants were clumped using R ld_clump function (ieugwasr R package) using clump_kb = 10,000, clump_r2 = 0.001, and clump_p = 0.05. The resulting variants showing BF > 1 were mapped to genes through Ensembl platform [45]. A Gene Ontology (GO) enrichment analysis was performed using the Database for Annotation, Visualization and Integrated Discovery (DAVID) [46]. GO terms surviving false discovery rate (FDR) multiple testing correction (*q* < 0.05) were retained as significant.

## Results

### SNP-based heritability and genetic correlation among hearing-problem traits assessed in the discovery cohorts

Because HP assessment was different among the cohorts included in the discovery GWAS meta-analysis (see “Cohorts and hearing-problem assessment”), we conducted SNP-h^2^ and genetic correlation among HP traits available from UKB, NHS, and HPFS. In UKB, multiple HP traits were available in the UKB (Table 1). These included self-reported HP phenotypes and an automated hearing assessment (i.e., SRT test). For the questionnaire-derived HP traits, the SNP-h^2^ ranged from 0.017 ± 0.002 for “Hearing aid use” to 0.050 ± 0.002 for “Hearing difficulties with background noise”. Because of the smaller sample size of NHS and HPFS cohorts, their HP SNP-h^2^ estimates (NHS: SNP-h^2^ = 0.077 ± 0.020, *z* = 3.85; HPFS SNP-h^2^ = 0.161 ± 0.053, *z* = 3.04) were less accurate than those from UKB (Table 1). No statistical differences were present between UKB female vs male or between NHS vs HPFS SNP-h^2^ estimates. The UKB genome-wide statistics of HP traits were characterized by inflation due to polygenicity (genomic-control lambda > 1.09) and not because of possible confounders (LDSC intercept < 1.03; Additional file 1: Table S3). The null or very low heritability of the SRT-derived traits (see Additional file 2) is in line with previous findings [11, 47]. Accordingly, they were excluded from further evaluations.

**Table 1:** SNP heritability (SNP-h^2^) of hearing problems assessed in the UK Biobank. Sample size (*N*) is reported as case/control and total sample size for binary and quantitative traits, respectively. *SNP-h2* SNP-based heritability, *SE* standard error

Analysis	Trait	*N*	SNP-h^2^	SE	SNP-h^2^ *Z*-score
Sex-combined	Hearing difficulty/problems	125,011/340,771	0.038	0.002	20.16
	Hearing difficulty/problems with background noise	183,497/292,534	0.050	0.002	26.11
	Hearing aid user	17,754/303,823	0.017	0.002	9.67
	Speech-reception-threshold (SRT) estimate (left)	195,188	0.002	0.002	0.96
	Speech-reception-threshold (SRT) estimate (right)	195,916	0.009	0.002	3.74
	Hearing problem ordinal trait	300,958	0.045	0.003	15.23
Males	Hearing difficulty/problems	68,781/145,768	0.048	0.003	15.45
	Hearing difficulty/problems with background noise	97,002/121,104	0.050	0.003	16.23
	Hearing aid user	9,882/146,135	0.021	0.003	6.59
	Hearing problem ordinal trait	147,788	0.051	0.004	12.73
Females	Hearing difficulty/problems	56,230/195,003	0.040	0.003	14.21
	Hearing difficulty/problems with background noise	86,495/171,430	0.054	0.003	18.79
	Hearing aid user	7,872/157,688	0.017	0.003	5.55
	Hearing problem ordinal trait	153,170	0.054	0.004	13.5

**Fig. 1:**
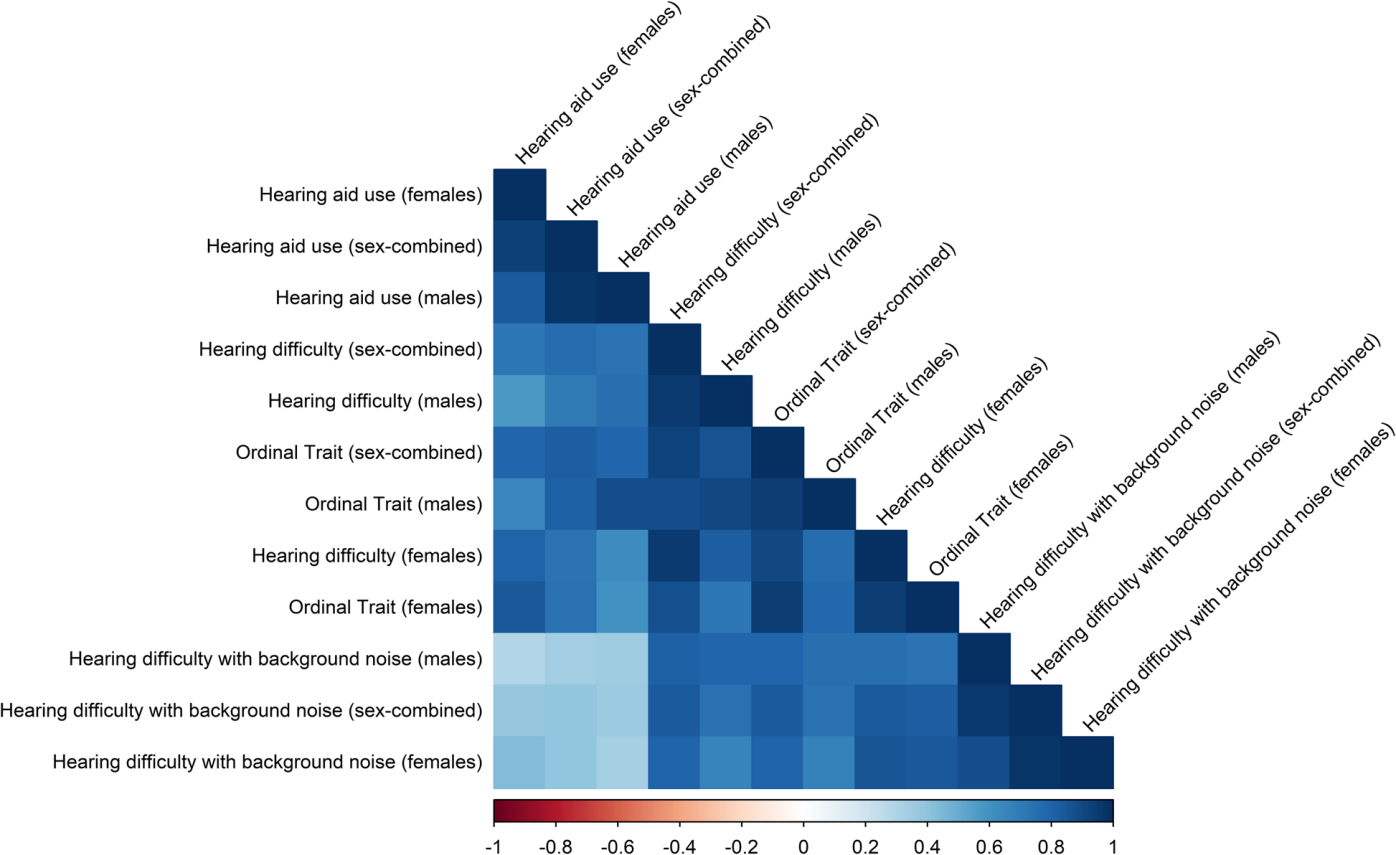
Genetic correlation among hearing-problem traits assessed via questionnaire in the UK Biobank. The square shade intensity is proportional to the magnitude of the correlation. Details regarding the results shown are available in Additional file 1: Table S4

The genetic correlations among the questionnaire-derived HP traits in UKB ranged from rg = 0.393 ± 0.039 between “Hearing difficulties with background noise” and “Hearing aid use” to rg = 0.830 ± 0.013 between “Hearing difficulties” and “Hearing difficulties with background noise” (Fig. 1; Additional file 1: Table S4). The ordinal phenotype derived from combining the three questionnaire-derived HP traits in UKB (Additional file 1: Table S1; hereafter abbreviated as HP-ORD) showed a high genetic correlation with each of the binary phenotypes, ranging from rg = 0.830 ± 0.020 with “Hearing aid use” to rg = 0.927 ± 0.015 with “Hearing difficulties”.

We also conducted a sex-stratified analysis, testing the genetic correlation among HP traits assessed in UKB and with HP phenotypes assessed in NHS (female participants only) and HPFS (male participants only). With respect to genetic correlation, “*Hearing difficulties with background noise*” showed the highest rg between males and females (rg = 0.882 ± 0.035), while the lowest rg between sexes was observed for the HP-ORD trait (rg = 0.782 ± 0.050). Considering the HPFS HP outcome and male-specific UKB analyses, the highest genetic correlation was observed for “Hearing difficulty” (rg = 0.633 ± 0.136). The same trait was the one with the highest genetic correlation with the NHS HP outcome among the UKB-female-specific analyses (rg = 0.737 ± 0.115). Based on the genetic correlations among UKB, HPFS, and NHS, the UKB “Hearing difficulties” was defined as the primary UKB phenotype. To maximize the discovery of our analyses, we meta-analyzed UKB, NHS, and HPFS cohorts (278,744 female participants from UKB and NHS and 223,081 male participants from UKB and HPFS).

### Genome-wide association meta-analysis

The HP GWAS meta-analysis of UKB, NHS, and HPFS (*N* = 501,825, 29% cases) identified 4382 genome-wide significant (GWS) variants related to 54 LD-independent loci (*P* < 5 × 10^−8^; LD *r*^2^ < 0.1) (Table 2; Additional file 2: Fig. S1). Nine of the 54 lead variants were available only in UKB. In the sex-stratified investigation, we gained one novel locus in the male-specific GWAS meta-analysis (15 LD-independent GWS loci) and five novel loci in the female-specific GWAS meta-analysis (24 LD-independent GWS loci). Few index variants identified in the sex-specific GWAS meta-analysis were LD-independent of the loci identified in the sex-combined GWAS meta-analysis. Additionally, we identified statistical differences in the sex-specific effects (calculated with a *z*-test; Bonferroni significance difference-*p* = 1.28 × 10^−3^) for four female-specific GWS variants (Additional file 1: Table S5): rs13399656 (female-beta =  − 0.058 vs. male-beta =  − 0.014, difference-*p* = 3.94 × 10^−5^), rs1808828 (female-beta = 0.047 vs. male-beta = 0.011, difference-*p* = 1.25 × 10^−4^), rs11738813 (female-beta = 0.063 vs. male-beta = 0.031, difference-*p* = 5.30 × 10^−4^), and rs35624969 (female-beta = -0.047 vs. male-beta = -0.012, difference-*p* = 1.22 × 10^−3^).

**Table 2:** LD-independent loci associated with hearing problems reaching genome-wide significance (*p* < 5 × 10^−8^) in the meta-analysis of UK Biobank (UKB), Nurses’ Health Studies (NHS; I and II), and Health Professional Follow-up Study (HPFS). The results of the replication analysis in Million Veteran Program (MVP) are also reported. *Abbreviations*: *CHR* chromosome, *POS* position, *bp* base pairs, *EAF* effect allele frequency, *OR* odds ratio, *P P*-value

rsID	CHR	POS (bp)	Effect allele	Other allele	UKB-NHS-HPFS meta-analysis		MVP replication	
					**OR**	***P***	**OR**	***P***
rs11073804	15	89,252,012	A	T	0.95	5.60 × 10^−17^	0.91	3.50 × 10^−28^
rs9493627	6	133,789,728	A	G	1.04	1.03 × 10^−17^	1.06	1.91 × 10^−19^
rs10901863	10	126,812,270	T	C	1.05	6.21 × 10^−23^	1.06	1.17 × 10^−11^
rs1566128	14	52,514,981	A	G	1.04	1.02 × 10^−15^	1.04	3.98 × 10^−11^
rs2254303	6	43,276,390	A	G	1.05	1.19 × 10^−23^	1.04	5.23 × 10^−11^
rs143282422	10	73,377,112	A	G	1.16	4.43 × 10^−11^	1.22	8.30 × 10^−11^
rs7819550	8	74,206,582	A	G	1.04	6.96 × 10^−10^	1.05	3.92 × 10^−9^
rs6662164	1	46,146,230	A	T	1.04	6.59 × 10^−17^	1.04	7.44 × 10^−9^
rs6871548	5	73,076,024	T	C	1.05	2.30 × 10^−25^	1.04	1.23 × 10^−8^
rs6545432	2	54,817,683	A	G	1.04	1.05 × 10^−15^	1.04	2.37 × 10^−8^
rs739138	22	38,122,462	A	G	0.95	4.70 × 10^−19^	0.96	6.21 × 10^−8^
rs67307131	11	118,480,223	T	C	0.96	3.12 × 10^−18^	0.97	2.83 × 10^−7^
rs36062310	22	50,988,105	A	G	1.15	2.75 × 10^−32^	1.08	4.80 × 10^−6^
rs11238325	7	50,853,151	T	C	1.04	1.88 × 10^−11^	1.04	5.65 × 10^−6^
rs11022697	11	13,178,285	C	G	0.97	1.17 × 10^−9^	0.97	1.15 × 10^−5^
rs7712395	5	2,559,538	T	C	1.05	3.64 × 10^−11^	1.04	6.45 × 10^−5^
rs6675438	1	165,112,224	A	T	1.03	8.82 × 10^−11^	1.03	1.62 × 10^−4^
rs2296506	6	158,507,981	A	G	1.03	1.70 × 10^−13^	1.02	3.36 × 10^−4^
rs2877561	3	121,712,051	A	C	1.04	1.62 × 10^−14^	1.03	4.95 × 10^−4^
rs12156228	8	141,701,299	T	G	0.97	3.67 × 10^−12^	0.97	6.31 × 10^−4^
rs222836	17	7,133,162	A	G	0.97	1.13 × 10^−11^	0.98	7.37 × 10^−4^
rs11085064	19	4,209,152	A	G	1.04	2.84 × 10^−9^	1.03	9.49 × 10^−4^
rs57167368	10	80,521,351	A	G	0.96	9.91 × 10^−11^	0.97	0.001
rs72622588	3	182,003,490	T	G	0.95	1.53 × 10^−16^	0.97	0.002
rs58919600	5	92,970,519	T	C	1.04	1.86 × 10^−9^	1.03	0.002
rs13171669	5	148,601,243	A	G	0.97	1.15 × 10^−12^	0.98	0.007
rs73204028	7	115,092,852	T	C	0.97	2.20 × 10^−8^	0.98	0.012
rs5800853	12	109,790,748	CAG	C	1.03	4.87 × 10^−9^	1.02	0.017
rs1171114	6	84,227,646	T	C	0.97	2.69 × 10^−10^	0.98	0.018
rs35094336	8	82,670,771	A	G	1.06	2.07 × 10^−11^	1.03	0.021
rs72930998	18	52,632,968	T	C	0.97	2.03 × 10^−10^	0.98	0.028
rs1928176	6	21,968,899	A	G	0.97	5.44 × 10^−9^	0.99	0.028
rs61734651	20	61,451,332	T	C	1.06	1.30 × 10^−8^	1.03	0.028
rs9530470	13	76,416,638	A	G	0.97	5.64 × 10^−9^	0.99	0.051
rs11041717	11	8,054,933	A	C	0.96	7.31 × 10^−15^	0.98	0.071
rs717973	2	208,091,112	A	G	0.97	4.61 × 10^−10^	0.99	0.072
rs8063057	16	53,812,433	T	C	1.03	1.64 × 10^−8^	1.01	0.081
rs2354376	7	138,487,145	A	C	1.03	3.17 × 10^−10^	1.01	0.1
rs78229182	6	151,128,784	T	C	1.05	2.07 × 10^−8^	0.98	0.106
rs148512269	2	227,596,034	T	G	1.11	9.83 × 10^−9^	1.05	0.133
rs72818515	10	76,060,718	A	C	1.03	7.08 × 10^−10^	1.01	0.186
rs111935448	16	30,919,807	T	C	1.04	1.75 × 10^−9^	0.99	0.218
rs35624969	7	72,991,592	T	C	0.97	4.66 × 10^−8^	0.99	0.218
rs4948502	10	63,839,417	T	C	1.03	1.26 × 10^−11^	1.01	0.229
rs566673	11	66,401,373	T	G	0.97	1.80 × 10^−8^	1.01	0.277
rs34073570	5	103,998,895	CT	C	0.97	1.32 × 10^−9^	0.99	0.316
rs112725535	1	6,494,086	A	G	0.96	7.85 × 10^−10^	0.99	0.318
rs2273654	10	102,689,217	T	C	0.97	4.01 × 10^−8^	0.99	0.425
rs8102051	19	2,370,476	A	C	1.03	3.38 × 10^−9^	1.01	0.429
rs13147559	4	17,524,570	C	G	0.95	3.81 × 10^−14^	0.99	0.596
rs61863078	10	94,777,108	A	G	1.03	1.54 × 10^−9^	1	0.777
rs766262445 (MVP LD-proxy: rs9536378)	13	53,830,039	CT(A)	C	1.03	2.63 × 10^−8^	1	0.787
rs34993346	11	89,046,097	A	G	0.95	1.38 × 10^−24^	1	0.872
rs9783279	11	68,972,992	A	C	0.97	2.97 × 10^−9^	1	0.872

Considering the 54 loci reaching genome-wide significance in the sex-combined discovery GWAS (i.e., the meta-analysis of UKB, NHS, and HPFS cohort), we tested whether the effects detected were replicated in an independent sample, 226,043 EUR participants from the MVP cohort. We observed that 34 loci were at least nominally replicated in the MVP cohort (Table 2; *p* < 0.05). Considering concordance between discovery and replication cohorts, 49 loci showed consistent effect direction. The probability of observing concordant directions of 49 or more loci out of the 54 loci tested by chance is 5.9 × 10^−6^. Leveraging our discovery GWAS meta-analysis as a training dataset, we conducted a PRS analysis in the MVP cohort that showed a small but highly statistically significant association (R^2^ = 0.54%, *p* = 1.78 × 10^−216^; Additional file 1: Table S6). Low but statistically significant predictive power was also observed when using UKB-only GWAS to perform a PRS analysis with respect to NHS-HPFS combined sample (R^2^ = 0.05%, *p* = 6.59 × 10^−6^; Additional file 1: Table S7). Compared with the sex-combined UKB-only PRS analysis, slightly higher predictive power was detected in the sex-stratified UKB-only PRS analyses (UKB-female PRS → NHS: R^2^ = 0.47%, *p* = 1.77 × 10^−30^; Additional file 1: Table S8; UKB-male PRS → HPFS: R^2^ = 0.50%, *p* = 2.84 × 10^−11^; Additional file 1: Table S9).

To assess the cross-ancestry generalizability of the loci identified by the GWAS meta-analysis, we conducted a replication analysis of the single-variant associations in the UKB non-EUR participants (AFR *N* = 6636, 11% cases; AMR *N* = 980, 16% cases; CSA *N* = 8876, 17% cases; EAS *N* = 2709, 12% cases; MID *N* = 1599, 16% cases). Considering FDR multiple testing correction (FDR *q* < 0.1), we replicated the associations of two variants: rs6662164 in CSA (OR = 1.14, *p* = 0.002) and rs34993346 in EAS (OR = 0.69, *p* = 0.001). Nominally significant replication was observed for other twelve variants (four in AFR, one in AMR, five in CSA, one in EAS, one in MID; Additional file 1: Table S10). The limited number of single-loci replication is due to the dramatic difference in sample size between the EUR discovery cohort (*N* = 501,825) and the non-EUR replication cohorts (total *N* = 20,800). However, we observed a significant cross-ancestry transferability in the HP PRS: AFR R^2^ = 4.73%, *p* = 4.65 × 10^−68^ (Additional file 1: Table S11; AMR R^2^ = 1.96%, *p* = 1.23 × 10^−5^ (Additional file 1: Table S12; CSA R^2^ = 3.5%, *p* = 6.26 × 10^−66^ (Additional file 1: Table S13; EAS R^2^ = 2.45%, *p* = 1.94 × 10^−15^ (Additional file 1: Table S14).

### Variant prioritization, fine-mapping, and multi-tissue transcriptome-wide association study

To translate genetic associations identified in our HP discovery GWAS (i.e., UKB-NHS-HPFS GWAS meta-analysis of “Hearing difficulties”) into information regarding potential causal genes associated with HP, we integrated different approaches ranging from positional mapping to imputation of genetically regulated transcriptomic variation.

First, we performed a fine-mapping analysis for each GWS locus. Based on the PIP of the 4382 SNP associations reaching GWS in the sex-combined GWAS meta-analysis, we identified 218 variants that are most likely to be causal (PIP > 30%; Additional file 3: Fig. S2). Considering a CADD score threshold of 10 (top 1% of pathogenic variants across the human genome), we further prioritized 24 variants that mapped to 18 unique genes (Additional file 1: Table S15). Some of them (e.g., *ARID5B*, *CTBP2*, and *FTO*) were previously identified as causal loci of Mendelian forms of deafness [6, 11]. Applying the same mapping strategy to the sex-stratified GWS loci, we identified 84 and 46 variants in the credible set for female- and male-specific analyses, respectively (Additional file 3: Figs. S3 and S4). These included six female-specific and two male-specific pathogenic variants (CADD score > 10). The sex-stratified mapping genes mostly overlapped with the ones identified in the sex-combined GWAS meta-analysis.

**Fig. 2:**
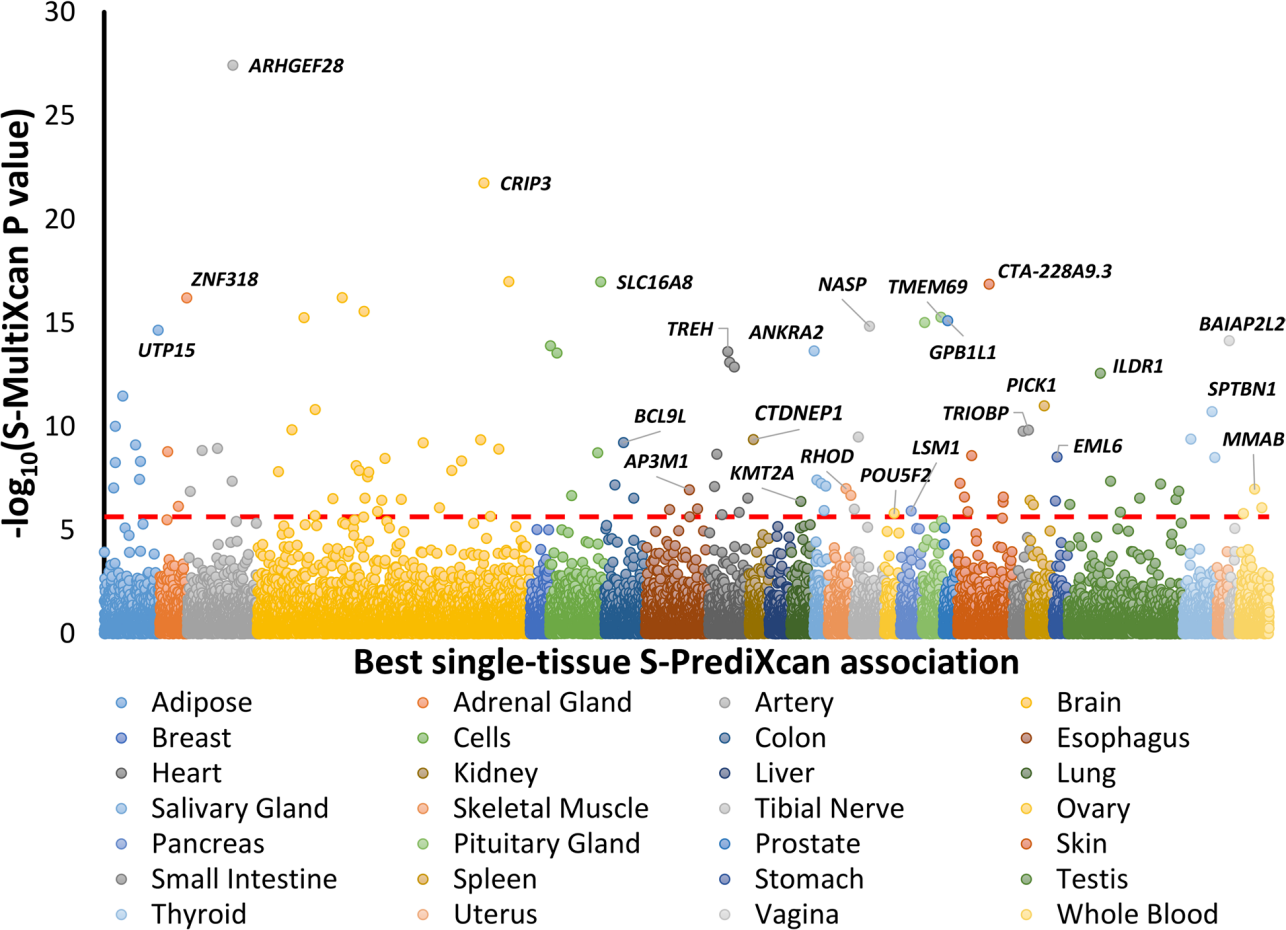
Multi-tissue transcriptome-wide association study of hearing problems based on the sex-combined meta-analysis. The *y*-axis corresponds to two-tailed − log10 (*p*-value of the S-MultiXcan association). The *x*-axis reports the genes grouped based on the best single-tissue S-PrediXcan association. The red line refers to the Bonferroni multiple testing correction accounting for the number of genes tested (*N* = 22,335; *p* < 2.24 × 10.^−6^). Bold labels are reported for the top-10 Bonferroni significant association. Additional labels are included for the top significant result for each tissue. Detailed results are available in Additional file 1: Table S16

In addition to the positional mapping approach, we also implemented an independent method based on the genetic regulation of transcriptomic variation. Combining tissue-specific information regarding expression quantitative trait loci (eQTL), we conducted a multi-tissue TWAS using the S-MultiXcan approach [41]. In the sex-combined meta-analysis, we identified 107 transcriptome-wide significant (TWS) genes (multi-tissue Bonferroni significance *p* < 2.24 × 10^−6^; Fig. 2; Additional file 1: Table S16).

Considering the top tissue-specific effect underlying the cross-tissue associations, we observed that five of the top-10 strongest associations were brain-related: putamen basal ganglia (*CRIP3 p* = 1.19 × 10^−23^), spinal cord c-1 (*PIK3R3 p* = 1.32 × 10^−19^), pituitary gland (*TMEM69 p* = 2.14 × 10^−18^), caudate basal ganglia (*IPP p* = 3.04 × 10^−18^), cerebellar hemisphere (*SLC22A7 p* = 3.56 × 10^−18^), and cerebellum (*MAST2 p* = 3.17 × 10^−17^). The strongest non-brain-related TWS associations included tibial artery (*ARHGEF28 p* = 3.29 × 10^−17^), cultured fibroblasts (*ACAN p* = 5.17 × 10^−17^), left ventricle (*DLK2 p* = 1.03 × 10^−16^), and tibial nerve (*NASP p* = 1.42 × 10^−16^). In the sex-stratified meta-analysis, we identified 55 and 26 multi-tissue TWS associations in females and males, respectively (Additional file 1: Table S17). The sex-specific loci mostly overlapped with those identified in the sex-combined TWAS: (i) 14 genes were TWS in three analyses; (ii) 45 out of 55 female-specific TWS genes were also significant in the sex-combined TWAS; (iii) 23 out of the 26 male-specific TWS genes were also significant in the sex-combined TWAS. However, for tissue-specific effects, we observed that the top genes were not related to brain transcriptomic regulation (top female-specific association: liver-*CRIP3 p* = 1.59 × 10^−18^; top male-specific association: spleen-*PHLDB1 p* = 1.59 × 10^−11^). Additionally, among female-specific associations, we identified associations related to female organs: mammary gland (*ABCC10 p* = 6.16 × 10^−15^) and vagina (*BAIAP2L2 p* = 7.45 × 10^−13^).

### Enrichment for regulatory elements and biological pathways

To dissect further HP polygenic architecture, we partitioned its SNP-h^2^ (estimated from UKB-NHS-HPFS GWAS meta-analysis of “Hearing difficulties”) with respect to regulatory elements to uncover enrichment of relevant biological annotations and processes. We observed that several annotations related to the regulatory function of the human genome are more likely to be involved in HP predisposition than that expected by chance (Additional file 1: Table S18). These included evolutionary-conserved regions (e.g., “genomic evolutionary rate profiling scores” *p* = 1.11 × 10^−16^) and “elements involved in epigenetic and transcriptomic regulation (e.g., “CpG dinucleotide content” *p* = 1.53 × 10^−10^; “super-enhancer regions” *p* = 1.54 × 10^−7^). In a complementary approach, we performed gene-set enrichment analysis of HP-associated genes (based on positional mapping). Two biological pathways were observed to be statistically overrepresented: “response to trabectedin” (*p* = 1.58 × 10^−8^) and “sensory perception of mechanical stimulus” (*p* = 1.69 × 10^−7^). With respect to both analyses, we did not identify statistical differences in the enrichments calculated from the sex-specific genetic associations.

### Phenome-wide genetic correlation and latent causal variable analysis of hearing problems

**Fig. 3:**
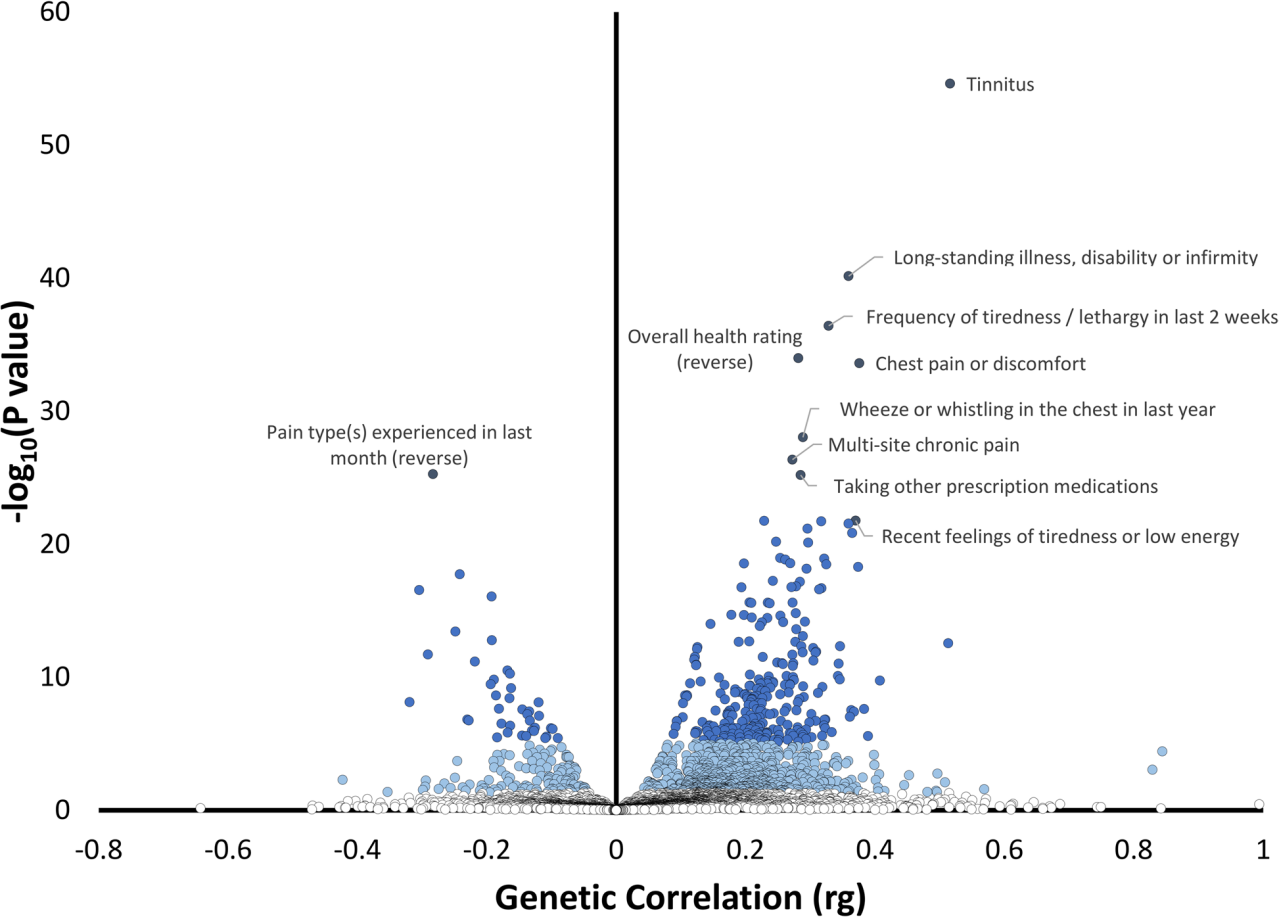
Phenome-wide genetic correlation of hearing problems in the sex-combined analysis. The *x*-axis reports the genetic correlation of hearing problems with the traits tested. The *y*-axis corresponds to two-tailed − log10(*p*-value). Blue shades correspond to significance strength, from white, non-significant (*p* > 0.05), to light blue (nominal significance *p* < 0.05), to blue (Bonferroni correction *p* < 6.99 × 10.^−6^), and dark blue (top 10 results). Phenotype labels are included for the top 10 results. Full results are reported in Additional file 1: Table S19

We analyzed the genetic correlation of HP with human traits and diseases, testing our HP discovery GWAS (i.e., UKB-NHS-HPFS meta-analysis) with respect to EUR genome-wide association statistics available from Pan-UKB analysis. Considering a Bonferroni correction accounting for the number of phenotypes tested in sex-combined, female, and male analyses (sex-combined *N* = 5337, *p* < 6.99 × 10^−6^; female *N* = 2,353, *p* < 1.52 × 10^−5^; male *N* = 2249, *p* < 1.59 × 10^−5^), we identified 309, 79, and 109 significant genetic correlations with HP, respectively (Fig. 3; Additional file 1: Table S19). In the sex-combined analysis, the strongest genetic correlation was “Tinnitus” (rg = 0.52, p = 2.44 × 10^−55^). Other strong positive genetic correlations included “Long-standing illness, disability or infirmity” (rg = 0.36, *p* = 7.12 × 10^−41^); and “Frequency of tiredness / lethargy in last 2 weeks” (rg = 0.33, *p* = 3.85 × 10^−37^). Among negative HP genetic correlations, we observed “Leisure/social activities” (rg =  − 0.19, *P* = 3.30 × 10^−10^) and “Belief that own life is meaningful” (rg =  − 0.18, *p* = 3.38 × 10^−6^). Although many of the sex-specific genetic correlations overlapped with those shared with sex-combined analysis, we also identified five traits with sex differences statistically significant after Bonferroni correction accounting for the number of phenotypes available in both female and male analyses (*N* = 1622; *p* < 3.08 × 10^−5^). Four of them were related to educational attainment with the strongest one observed for “Qualification: College or University degree” (female rg = 0.11, *P* = 2.52 × 10^−5^, male rg =  − 0.12, *P* = 1.15 × 10^−5^; *p*_sex-difference_ = 1.16 × 10^−9^). The fifth genetic correlation was related to “Time spend outdoors in summer” (female rg =  − 0.16, *P* = 2.73 × 10^−5^, male rg = 0.06, *P* = 0.08; *p*_sex-difference_ = 1.95 × 10^−5^).

**Fig. 4:**
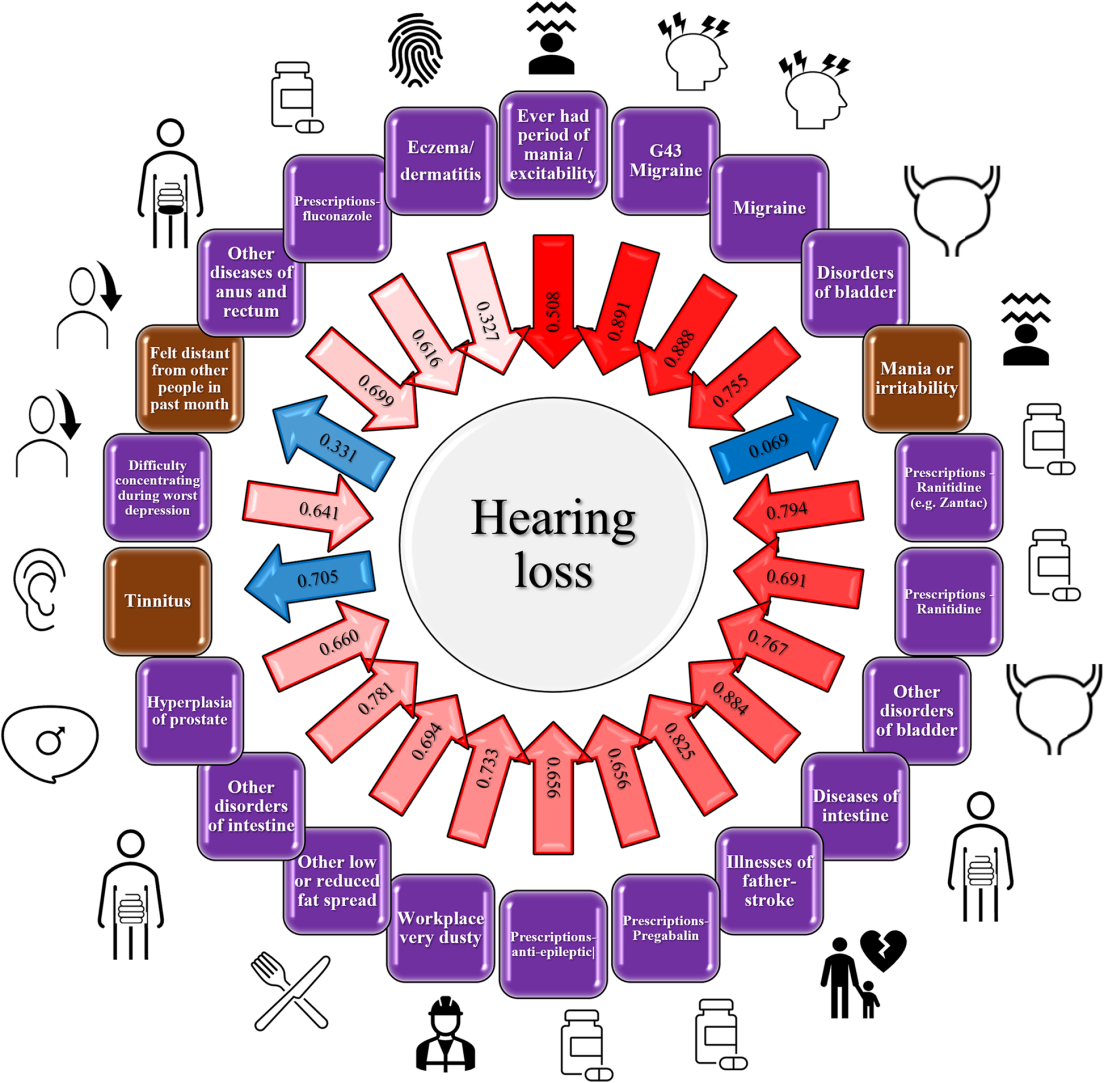
Visual representation of the 22 Bonferroni-significant putative causal effects identified through the latent causal variable analysis. Brown labels: HP has causative effect on the trait in the label. Purple labels: Trait in the label has causative effect on HP. The absolute gcp (genetic causality proportion) value for each association is reported within the arrow, and the directions refer to the cause-effect relationship (Blue: HP causes Trait; Red: Trait causes HP). The shade intensity of the arrows is proportional to the statistical significance (i.e., − log_10_(*p*-value)) of the gcp estimates. A description of each trait and details of the associations are available in Additional file 1: Table S20

To distinguish genetic correlations due to shared genetic mechanisms from those due to possible cause-effect relationships, we conducted an LCV analysis, identifying 22 significant putative causal effects after Bonferroni multiple testing correction (*p* < 5.69 × 10^−5^; Fig. 4, Additional file 1: Table S20). As mentioned in the methods, in the LCV analysis, positive and negative gcp values reflect the direction of the putative causal effect (i.e., HP → phenotype and phenotype → HP, respectively) while the sign of the effect is given by the rho statistics. The traits with putative causal effect on HP included psychiatric traits (e.g., “Ever had period of mania / excitability” gcp =  − 0.508, *p* = 3.59 × 10^−23^; rho = 0.275, SE = 0.075), neurological disease (e.g., “Migraine” gcp =  − 0.891, *p* = 7.1 × 10^−21^; rho = 0.272, SE = 0.072), gastrointestinal outcomes (e.g., “ICD-10 K63 Other diseases of intestine” gcp =  − 0.883, p = 2.25 × 10^−11^; rho = 0.226, SE = 0.070), urogenital outcomes (e.g., “ICD-10 N32 Other disorders of bladder” gcp =  − 0.755, *p* = 7.83 × 10^−15^; rho = 0.212, SE = 0.084), medication use (e.g., “Ranitidine” gcp =  − 0.691, *p* = 1.38 × 10^−12^; rho = 0.273, SE = 0.074), cardiovascular diseases (“Stroke family history” gcp =  − 0.825, *p* = 6.67 × 10^−11^; rho = 0.303, SE = 0.075), immunological conditions (“Eczema/dermatitis” gcp =  − 0.327, *p* = 3.82 × 10^−5^; rho = 0.103, SE = 0.056), and workplace environment (“Workplace very dusty” gcp =  − 0.733, *p* = 2.04 × 10^−10^; rho = 0.235, SE = 0.057). We also identified genetic evidence that supports a putative causal role on HP for three outcomes: “Tinnitus” (gcp = 0.705, *p* = 4.85 × 10^−8^; rho = 0.519, SE = 0.038); “Manifestations of mania or irritability” (gcp = 0.069, *p* = 8.44 × 10^−15^; rho = 0.357, SE = 0.075), and “Felt distant from other people in past month” (gcp = 0.331, *p* = 6.52 × 10^−6^; rho = 0.232, SE = 0.074). No gcp estimate showed a statistically significant difference between sexes after multiple testing correction.

### Multivariate gene-by-environment genome-wide interaction analysis

To expand our understanding of possible interactive effects beyond those already observed in the sex-stratified GWAS, we conducted a multivariate GEWIS exploring variables related to noise pollution and tobacco smoking, well-known HP risk factors [13–15]. Because of the assumptions of the linear mixed model approach used [44], we conducted the GEWIS, testing HP-ORD trait (UKB *N* = 300,818) with respect to 14 environmental factors simultaneously (Additional file 1: Table S2). In addition to sex, these include environments related to noise pollution (*N* = 5) and tobacco smoking habits (*N* = 8). The GEWIS was conducted using the structLMM approach, which is not affected by the degree of correlation among the environmental factors investigated [44]. Although no result survived genome-wide testing correction, we identified 1278 LD-independent variants with nominally significant multivariate interactions (Additional file 1: Table S21) that were enriched for 57 unique GO terms after FDR multiple testing correction (FDR *q* < 0.05; See Additional file 2). The strongest enrichments were observed for GOs related to neurodevelopmental processes (e.g., GO:0,007,399 Nervous system development: “Sex” *p* = 2.0 × 10^−6^; “Average evening sound level of noise pollution” *p* = 1.3 × 10^−6^; “Maternal smoking around birth” *p* = 3.6 × 10^−7^).

## Discussion

We conducted a large-scale investigation integrating information from genome-wide associations with tissue-specific transcriptomic variation and a genetically informed causal inference analysis to translate genetic findings into insights regarding HP biology and epidemiology. Initially, we compared the SNP-h^2^ of different HP definitions available from UKB. These included three questionnaire-derived self-reported hearing traits and one trait derived from audiometric measures. Consistent with the missing heritability observed for complex traits [48], we observed that the SNP-based heritability of HP traits (up to 5%; in line with previous HP GWAS; [11, 47]) was much lower than the heritability estimated by previous family-based analyses (30–70%) [6]. Nevertheless, self-reported HP outcomes showed significant SNP-h^2^ that was informative to investigate their polygenic architecture. This is in line with previous studies that showed self-reported HP traits appear to be reliable instruments to investigate “real-world” hearing impairment [49–53], although they may still be potentially influenced by psychosocial factors [11]. Conversely, in agreement with previous studies [11, 47], we observed a null SNP-h^2^ for the trait based on the audiometric measures. Further studies are needed to understand the potential issues of assessments based on audiometric tests.

We observed a high genetic correlation (rg > 0.7) between self-reported HP outcomes with the only exception being “Hearing difficulty with background noise” and “Hearing aid use” where the genetic correlation was much lower (rg < 0.4). This may be because the proportion of individuals who seek hearing aids for their HP is very low, even in the UK where hearing aids are covered by the National Health Service. Considering the HP outcome available in NHS and HPFS cohorts, we observed the highest correlation with the UKB outcome “Hearing difficulties”. Accordingly, these traits were used for the GWAS meta-analysis and the subsequent in silico investigations.

In the sex-combined GWAS meta-analysis including 501,825 individuals, we identified 54 LD-independent GWS associations, 12 of which are novel with respect to those identified by previous HP GWAS [6, 9, 11, 54, 55]. Although the cohorts included in our discovery GWAS (i.e., UKB, NHS, and HPFS) were recruited and assessed using different strategies, we did not identify heterogeneity among the cohort-specific effects. However, cross-cohort PRS associations presented a very small predictive power likely due to differences in the cohort characteristics. A similar situation was also present with respect to the MVP cohort (i.e., good single-variant replication rate but very small predictive power in the cross-cohort PRS association).

Although this primary analysis was conducted in EUR individuals, we also investigated 20,800 UKB participants of AFR, AMR, CSA, EAS, and MID descent. Due to the limited non-EUR sample, we replicated only few single-variant associations and none of them were present across multiple ancestries. While the low statistical power likely played a key role in the lack of multiple-ancestry single-variant replications, differences in allele frequency and LD structure may also have contributed [56]. Conversely, we observed highly statistically significant associations of HP PRS derived from the EUR discovery GWAS meta-analysis and tested on non-EUR participants. Consistent with previous studies of other health outcomes [57–59], there were large differences in the predictive power due to the genetic diversity across human populations. Additionally, in some cases, the HP variance explained by the cross-ancestry PRS association (e.g., EUR-PRS_UKB-NHS-HPFS_ → AFR-UKB R^2^ = 4.73%) is larger than the one observed in the same-ancestry PRS association (e.g., EUR-PRS_UKB-NHS-HPFS_ → EUR-MVP R^2^ = 0.54%). This can be explained by the fact that the cross-ancestry results are based on the UKB cohort where the participants share the same HP assessment and sample characteristics. However, we cannot exclude that the different LD structures of the ancestries investigated may have partially contributed to inflate the results observed.

The fine-mapping of the risk loci led to identifying putatively pathogenic variants (CADD score > 10) mapped to genes previously demonstrated to be involved in HP pathogenesis (see Additional file 2). Our TWAS further highlighted that HP genetic basis is partially linked to brain transcriptomic regulation. We identified several genes previously implicated in HP pathogenesis because of their potential involvement in the peripheral structures of the auditory system, which also showed predicted expression differences in various brain regions (see Additional file 2). A recent study investigated single-cell RNA-sequencing data from mouse cochlea and brain, mapping common variants associated with HP to spindle, root, and basal cells from the stria vascularis, a structure in the cochlea necessary for normal hearing [55]. We also leveraged the genome-wide association statistics generated by our analyses to explore HP polygenic architecture in the context of other traits and diseases. Our phenome-wide genetic correlation analysis identified a wide range of health outcomes that share a significant proportion of their genetic liability with HP. The subsequent genetically informed causal inference analysis showed that some of these genetic correlations may be due to cause-effect relationships linking HP to different health domains, including neurological, cardiovascular, and cancer-related outcomes (see Additional file 2). With respect to gene-environment interactions, although our multivariate GEWIS analysis identified FDR-significant enrichments for several brain-several biological pathways (see Additional file 2), no single interactive locus survived a genome-wide multiple testing correction. This supports that discovery analyses of HP gene-environment interaction may require a larger sample size.

With respect to sex differences, our UKB-NHS-HPFS GWAS meta-analysis identified three variants with statistical differences between sexes in their effect size. Specifically, rs13399656 in *SPTBN1* was the top finding in the sex-difference associations. This gene was previously recognized as a target for β-estradiol as the top upstream regulator [60]. Similarly, rs1808828 mapped to *ABLIM3* and rs11738813 in *ARHGEF28* were identified as intronic variants mapped in genes involved in estrogen signaling pathway [61, 62]. These sex-specific associations in significant loci involved in hormonal regulating pathways may reflect the potential role of estrogen on hearing functions. In line with these findings, our sex-stratified TWAS showed several additional associations related to peripheral tissues. In particular, the female-specific TWAS identified several transcriptomic changes related to breast mammary tissue (*ABCC10*) and vagina (*BAIAP2L2*). These associations in hormonally regulated tissues may reflect a potential role of estrogen in hearing function [63]. Indeed, our sex-stratified analyses identified associations mapped in genes involved in the estrogen signaling pathway (i.e., *SPTBN1*, *ABLIM3*, and *ARHGEF28*). With respect to the loci identified by the female-specific TWAS, *BAIAP2L2* is particularly interesting. In mice, mutations of the homologous gene Baiap2l2 were associated with alterations in hair cell transduction and deafness while the human locus *BAIAP2L2* is associated with suppression of the estrogen-mediated S–phase entry pathway in cell cycle [64]. As HP is associated with reduced estrogen levels, we hypothesize that the interplay between *BAIAP2L2* transcriptomic regulation and estrogen levels may play a role in HP in women. With respect to HP pleiotropy, our genetic correlation analysis also identified five traits that showed statistically significant sex differences in their correlation with HP. Four of them were related to educational attainment where the genetic correlation with HP was positive in females and negative in males. A previous study demonstrated that the characteristics and the recruitment strategy of the UKB cohort (the largest sample in our meta-analysis) influenced some of the sex differences observable in this study population [65]. In particular, higher educational attainment (EA) was genetically correlated with female sex in UKB, but an opposite relationship was observed in cohorts that used different recruitment strategies. Accordingly, the sex differences observed with respect to educational attainment may be specific to the structure of the UKB cohort. The other sex difference was related to time spent outside that showed a negative genetic correlation in females but not in males. This could be related to the higher comorbidity of HP with depression observed in women compared to men [66].

Although our findings advance our understanding of HP and its consequences in adults, we also acknowledge several limitations. Similar to previous HP GWAS [6, 9–11, 54, 55], our primary analyses were based on self-reported data. These appear to be more informative than the audiometric measures derived from SRT test, but they can still be biased by misreporting linked to cognitive processes, social desirability, and survey conditions. Our study was focused on acquired HP in adults and we excluded individuals with congenital HP when possible. However, we did not have information regarding HP age of onset across all cohorts investigated. Accordingly, a small proportion (< 1%) of congenital HP cases may be present in our study populations. We used MVP as our primary replication. Although we observed a good single-variant replication rate and highly statistical PRS associations, the sex unbalance present in this cohort (93% male participants) may be responsible for the limited predictive power of the PRS derived from our discovery GWAS (i.e., UKB-NHS-HPFS meta-analysis), where sexes are more equally represented. No sex-specific TWAS models are currently available from GTEx. Accordingly, our sex-stratified TWAS was conducted using sex-stratified genome-wide association statistics and sex-combined transcriptomic reference panels. This has likely reduced the statistical power of our analysis. Additionally, GTEx does not include tissues related to peripheral auditory systems, limiting our ability to explore the differences in transcriptomic changes in the peripheral and central auditory system. Although we showed highly statically significant PRS association across multiple ancestry groups, the limited diversity of the cohorts investigated did not permit us to explore the genetic basis of HP across ancestry groups and provided us with very limited statistical power to replicate single-variant associations. Further studies of more diverse populations are needed to conduct powerful gene discovery analyses of HP across ancestry groups. Similarly, our genetic correlation and genetically informed causal inference analyses identified several aging-related outcomes. Because our analyses were based on GWAS datasets that included covariates to account for age-related effects, we believe that these effects are due to the impact of HP on human health rather than age-related differences. However, we cannot exclude that there may be differences across different age groups. Unfortunately, the sample size available did not provide us with the statistical power needed to conduct phenome-wide genetic correlation analyses across multiple age groups.

## Conclusions

We conducted a comprehensive investigation of the polygenic architecture of HP in adults that (i) identified novel risk loci, (ii) provided evidence of the shared HP pathogenesis across human populations, (iii) integrated genetic and transcriptomic data to dissect HP biology, (iv) leveraged genome-wide information to explore the mechanisms underlying HP comorbidities, and (v) uncovered possible biological processes that could underlie inter-individual differences in susceptibility to the effects of HP environmental risk factors. In particular, our sex-specific analyses and transcriptomic associations highlighted molecular pathways that may be targeted for drug development or repurposing. Additionally, the potential causal relationships identified may support novel preventive screening programs to identify individuals at risk.

## Supplementary Information

**Additional file 1: Table S1.** Definition of the combined four-category ordinal phenotype based on the traits defined in the UKB. Binary definitionswere combined to define a severity/probability scalAQe of hearing problems. **Table S2.** Environments investigated in the GEWIS analysis. **Table S3.** Inflation statistics of hearing problem genome-wide association studies. **Table S4.** Genetic correlations among the HP traits assessed in UKB considering sex-combined and and sex-stratified analyses. rg: genetic correlation; se: standard error. **Table S5.** Effect size comparison between sex-specific findings in the meta-analyses. Results surviving multiple testing correction are highlighted in red. GWS:genome-wide significance in; REF: reference allele; ALT: alternative allele; A1:Effect allele; SE:standard error. **Table S6.** Association of the hearing-problem polygenic risk score derived from the UKB-NHS-HPFS sex-combined meta-analysis in the MVP cohort. **Table S7.** Association of the hearing-problem polygenic risk score derived from the UKB-only sex-combined GWAS in the NHS-HPFS combined sample. **Table S8.** Association of the hearing-problem polygenic risk score derived from the UKB-only female-specific GWAS in the NHS sample. **Table S9.** Association of the hearing-problem polygenic risk score derived from the UKB-only male-specific GWAS in the HPFS sample. **Table S10.** Single-variant replication in other ancestry groups available in UKB. REF: reference allele; ALT: alternative allele; se:standard error; pval:P; af:allele frequency. FDR significantand nominally significant replications are highlighted in yellow and red, respectively. **Table S11.** Association of the hearing-problem polygenic risk score derived from the UKB-NHS-HPFS sex-combined meta-analysis in the UKB AFR sample. **Table S12.** Association of the hearing-problem polygenic risk score derived from the UKB-NHS-HPFS sex-combined meta-analysis in the UKB AMR sample. **Table S13.** Association of the hearing-problem polygenic risk score derived from the UKB-NHS-HPFS sex-combined meta-analysis in the UKB CSA sample. **Table S14.** Association of the hearing-problem polygenic risk score derived from the UKB-NHS-HPFS sex-combined meta-analysis in the UKB EAS sample. **Table S15.** Prioritized genes considering a CADD score threshold of 10. MAF:Minor allele frequency; se: standard error. **Table S16.** HP-associated genesin the Multi-tissue TWAS using S-MultiXcan. **Table S17.** Multi-tissue TWS associations after Bonferroni multiple testing correction identified in the sex-stratified analyses. **Table S18.** Enrichment for specific genomic features with respect to sex-combined and sex-stratified analyses. Results surviving Bonferroni multiple testing correction are highlighted in yellow. **Table S19.** Phenome-wide genetic correlation with HP considering sex-combined GWAS. Results surviving multiple testing correction are highlgihted in red rg: genetic correlation; se:standard error. **Table S20.** LCV analysis for HP. Results surviving Bonferroni multiple testing correction are indicated in red. gcp p: p-value for the genetic causality proportion; gcp: posterior genetic causality proportion; gcp se: standard error for the genetic causality proportion estimate; rho.est: genetic correlation estimate from LCV; rho.err: standard error for genetic correlation estimate from LCV. **Table S21.** Environment-specific Bayes factors of the LD-independent variants with nominally significant multi-environment interactions.

**Additional file 2.** Contains Supplementary Results and Supplementary Discussion.

**Additional file 3: Figure S1.** Manhattan plot of the sex-combined GWAS meta-analysis of UKB, NHS, and HPFS cohorts. The red line refers to the genome-wide significance threshold. **Figure S2.** Visual representation of the fine-mapping analysis for sex-combined meta-analysis. Each panel refers to a GWS risk locus. The variants were identified according to their LD with respect to the lead SNP and inclusion in the credible set with at most ten causal variants. **Figure S3.** Visual representation of the fine-mapping analysis for female meta-analysis. Each panel refers to a GWS risk locus. The variants were identified according to their LD with respect to the lead SNP and inclusion in the credible set with at most ten causal variants. **Figure S4.** Visual representation of the fine-mapping analysis for male meta-analysis. Each panel refers to a GWS risk locus. The variants were identified according to their LD with respect to the lead SNP and inclusion in the credible set with at most ten causal variants.

## Data Availability

The genome-wide association statistics generated by the current study are available in Zenodo, 10.5281/zenodo.7897038.

## Abbreviations

AFR: African
AMR: Admixed-American
BF: Bayes factor
CADD: Combined annotation dependent depletion
CSA: Central/South Asian
DAVID: Database for annotation, visualization and integrated discovery
EAS: East Asian
eQTL: Expression quantitative trait loci
FDR: False discovery rate
FUMA: Functional mapping and annotation of genome-wide association studies
gcp: Genetic causality proportion
GEWIS: Gene-by-environment genome-wide interaction study
GO: Gene Ontology
GTEx: Genotype-Tissue Expression
GWAS: Genome-wide association studies
GWS: Genome-wide significant
HARE: Harmonized ancestry and race/ethnicity
HP: Hearing problems
HP-ORD: Ordinal phenotype derived from combining the three questionnaire-derived HP traits in UKB
HPFS: Health Professionals Follow-up Study
LCV: Latent causal variable
LD: Linkage disequilibrium
LDSC: Linkage disequilibrium score regression
LoF: Loss-of-function
MAF: Minor allele frequency
MAGMA: Multi-marker analysis of genomic annotation
MID: Middle East
MVP: Million veteran program
NHS: Nurses’ health studies
PCs: Principal components
PIP: Posterior inclusion probability
PRS: Polygenic risk score
rg: Genetic correlation
SNP: Single-nucleotide polymorphism
SNP-h^2^: SNP-based heritability
SRT: Speech Recognition Threshold
TWAS: Transcriptome-wide association study
TWS: Transcriptome-wide significant
UKB: UK Biobank
WHO: World Health Organization
