# Supplementary material for: Sex differences in the polygenic architecture of hearing problems in adults
Source: Genome Med. 2023 May 11;15:36. doi: 10.1186/s13073-023-01186-3 (PMC10173489; doi:10.1186/s13073-023-01186-3)
Supplement: Supplementary file 2 — Additional file 2. Contains Supplementary Results and Supplementary Discussion. [file 13073_2023_1186_MOESM2_ESM.docx]

**ADDITIONAL FILE 2**

**Speech Recognition Threshold (SRT) – Supplementary Results**

With respect to STR-derived traits, the SNP-h^2^ was lower <1% with only right-ear assessment with an estimate statistically different from zero (SRT-right SNP-h^2^= 0.009±0.002; Table 1). Consistent with previous studies (1, 2), we observed that SRT-derived assessment had a very low SNP-h^2^ (<1%) that did not permit us to investigate further this HP outcome.

**Multivariate gene-by-environment genome-wide interaction analysis – Supplementary Results**

To identify environment-specific effects underlying the multivariate interactions of each of the 1,278 LD-independent variants with nominally significant multivariate interactions, we considered BF>1 following StructLMM reccomendations (3). We then conducted a GO enrichment analysis stratifying the LD-independent loci with nominally significant multivariate interactions based on the environment where they showed a BF>1. Considering FDR multiple testing correction at 5%, we identified 204 significant enrichments with respect to. Most GO enrichments were shared across loci showing interaction effects with specific sex, noise, and smoking environmental factors (BF>1). Specifically, loci with environment-specific interactions related to sex, noise, and smoking showed 17 shared GO enrichments with respect to (e.g., GO:0007399 Nervous system development: “S*ex”* p=2.0×10^-6^; *“Average evening sound level of noise pollution”* p=1.3×10^-6^; *“Maternal smoking around birth”* p=3.6×10^-7^). Similarly, loci interacting only with noise and smoking environment (but not sex) shared 17 GO enrichments (e.g., GO:0030182 Neuron differentiation: *“Noisy workplace”* p=4.4×10^-5^; *“Ever smoked”* p=4.4×10^-6^) and two GO enrichments related to loci with environment-specific interactions related to sex and smoking (e.g., GO:0050767 Regulation of neurogenesis: “*Sex”* p=7.5×10^-5^; *“Maternal smoking around birth*” p=4.6×10^-5^). We also identified enrichments related to loci interacting only with a specific environment category: seven GO terms related to sex interactions (e.g., GO:0030857 Negative regulation of cell differentiation: *“Sex”* p=8.2×10^-5^), 10 GO terms related to noise-pollution interactions (e.g., GO:0050885 Neuromuscular process controlling balance: *“Noisy workplace”* p=3.7×10^-4^), and three GO terms related to smoking interactions (e.g., GO:0016101 Diterpenoid metabolic process: *“Smoking/smokers in household”* p=2.1×10^-4^).

**Fine-mapping – Supplementary Discussion**

We identified the exonic variant rs13147559 mapping in *CLRN2* as the strongest finding (CADD=22.5). This locus has been identified as causative for non-syndromic HP (4) and may have a role in hair cell mechano-transduction (5). Two exonic mutations in *NOL12:TRIOBP* were also identified as potentially deleterious (rs5756795 and rs7284476). *TRIOBP* alterations are related to both congenital deafness (6) or Mendelian HL (7). Mutations mapped to this gene have been thoroughly analyzed, especially in patients with a post-lingual HL (i.e., HL occurring after the development of normal speech) (8,9). Non-coding regulatory variants were also identified in HL-related genes such as *DLG4, ARID5B,* and *CTBP2.* In mice, elevated Arid5b  in the aged cochlea is known to be critical for the etiologies of sensorineural HL, such as age-related HL, noise- and ototoxic drug-induced HL (10). Conversely, *DLG4* was previously identified as significantly related to hearing due to its association with the hippocampal glutamatergic synapse pathways (11). The deleterious variant mapping at *CTBP2* also supports the role of the central nervous system in HL pathogenesis. Indeed, CtBP2 is a marker for cochlear ribbon synapses, and deleterious variants (such as rs183893500, CADD=15.24) could influence vulnerability to cochlear synaptopathy (i.e., loss of nerve connections between the sensory cells and the central nervous system) in acquired sensorineural HL (12,13).

**TWAS and brain transcriptomic regulation – Supplementary Discussion**

While *CRIP3* has broad expression in inner and outer hair cells (14), we observed that transcriptomic changes of this gene in the putamen basal ganglia were associated with HL. Similarly, other genes that may be implicated in HP pathogenesis through both peripheral and central mechanisms include *IPP* (previously reported as highly expressed in human cochlear, cochlear hair cells, and spiral ganglion cells in mice (15)), *PIK3R3* (previously identified as specific to cell types in the cochlea (16)), *MAST2* (showing differential expression in the development of the inner ear (15)), and *SLC22A6* (identified as related to cochlear impairment (17)). Taken together, these results suggest that auditory dysfunction is not restricted to peripheral auditory structures, but subcortical neuroplastic changes involving the area receiving ascending signals from inferior brainstem nuclei could also be involved (18). Additionally, several TWAS associations were related to the transcriptomic regulation in the cerebellum in line with the involvement of this brain region in auditory pathways (19).

**Genetic Correlation and Causal Inference analysis – Supplementary Discussion**

In line with the shared involvement of the auditory system, the strongest HP genetic correlation was with tinnitus (i.e., the conscious perception of an auditory sensation in the absence of a corresponding external stimulus (20)). This is consistent with the strong association between tinnitus and HP in the elderly (21). A previous Mendelian randomization analysis showed a bidirectional relationship between them (22). However, our LCV analysis supports that HP genetic liability may have a causal effect on tinnitus. Further studies will be needed to understand the underlying dynamics of these comorbidities. Additionally, we observed genetic correlation with several traits related to the psychological distress generally observed in elderly individuals. They included positive genetic correlations with disability/infirmity and tiredness/lethargy and negative genetic correlations with the propensity to social activities and the belief that life is meaningful. These findings illustrate that HP is associated with multiple aspects of quality of life in aging populations.

**References**

1. Wells HRR, Freidin MB, Zainul Abidin FN, Payton A, Dawes P, Munro KJ, et al. GWAS Identifies 44 Independent Associated Genomic Loci for Self-Reported Adult Hearing Difficulty in UK Biobank. Am J Hum Genet. 2019;105(4):788–802.

2. Cherny SS, Livshits G, Wells HRR, Freidin MB, Malkin I, Dawson SJ, et al. Self-reported hearing loss questions provide a good measure for genetic studies: a polygenic risk score analysis from UK Biobank. Eur J Hum Genet. 2020;28(8):1056–65.

3. Moore R, Casale FP, Jan Bonder M, Horta D, Franke L, Barroso I, et al. A linear mixed model approach to study multivariate gene-environment interactions. Nat Genet. 2019;51(1):180–6.

4. Vona B, Mazaheri N, Lin SJ, Dunbar LA, Maroofian R, Azaiez H, et al. A biallelic variant in CLRN2 causes non-syndromic hearing loss in humans. Hum Genet. 2021;140(6):915–31.

5. Dunbar LA, Patni P, Aguilar C, Mburu P, Corns L, Wells HR, et al. Clarin‐2 is essential for hearing by maintaining stereocilia integrity and function. EMBO Mol Med. 2019;11(9):e10288.

6. Zhou B, Yu L, Wang Y, Shang W, Xie Y, Wang X, et al. A novel mutation in TRIOBP gene leading to congenital deafness in a Chinese family. BMC Med Genet. 2020;21(1):121.

7. Kalra G, Milon B, Casella AM, Herb BR, Humphries E, Song Y, et al. Biological insights from multi-omic analysis of 31 genomic risk loci for adult hearing difficulty. PLOS Genet. 2020;16(9):e1009025.

8. Kochhar A, Hildebrand MS, Smith RJH. Clinical aspects of hereditary hearing loss. Genet Med. 2007;9(7):393–408.

9. Pollak A, Lechowicz U, Murcia Pieńkowski VA, Stawiński P, Kosińska J, Skarżyński H, et al. Whole exome sequencing identifies TRIOBP pathogenic variants as a cause of post-lingual bilateral moderate-to-severe sensorineural hearing loss. BMC Med Genet. 2017;18:142.

10. Fujimoto C, Yamasoba T. Mitochondria-Targeted Antioxidants for Treatment of Hearing Loss: A Systematic Review. Antioxidants. 2019;8(4):109.

11. Deng T, Li J, Liu J, Xu F, Liu X, Mi J, et al. Hippocampal Transcriptome-Wide Association Study Reveals Correlations Between Impaired Glutamatergic Synapse Pathway and Age-Related Hearing Loss in BXD-Recombinant Inbred Mice. Front Neurosci. 2021;15:1356.

12. Liberman MC, Kujawa SG. Cochlear synaptopathy in acquired sensorineural hearing loss: Manifestations and mechanisms. Hear Res. 2017;349:138–47.

13. Xiong W, Yu S, Liu K, Gong S. Loss of cochlear ribbon synapses in the early stage of aging causes initial hearing impairment. Am J Transl Res. 2020;12(11):7354–66.

14. Praveen K, Dobbyn L, Gurski L, Ayer AH, Staples J, Mishra S, et al. Population-scale analysis of common and rare genetic variation associated with hearing loss in adults. Genetic and Genomic Medicine; 2021 http://medrxiv.org/lookup/doi/10.1101/2021.09.27.2126409163

15. Nagtegaal AP, Broer L, Zilhao NR, Jakobsdottir J, Bishop CE, Brumat M, et al. Genome-wide association meta-analysis identifies five novel loci for age-related hearing impairment. Sci Rep. 2019;9:15192. 79

16. Xue N, Song L, Song Q, Santos-Sacchi J, Wu H, Navaratnam D. Genes related to SNPs identified by Genome-wide association studies of age-related hearing loss show restriction to specific cell types in the adult mouse cochlea. Hear Res. 2021;410:108347.

17. Fitzakerley JL, Trachte GJ. Genetics of guanylyl cyclase pathways in the cochlea and their influence on hearing. Physiol Genomics. 2018;50(9):780–806.

18. Qu H, Tang H, Pan J, Zhao Y, Wang W. Alteration of Cortical and Subcortical Structures in Children With Profound Sensorineural Hearing Loss. Front Hum Neurosci. 2020;14:534.

19. Llano DA, Kwok SS, Devanarayan V, The Alzheimer’s Disease Neuroimaging Initiative (ADNI). Reported Hearing Loss in Alzheimer’s Disease Is Associated With Loss of Brainstem and Cerebellar Volume. Front Hum Neurosci. 2021;15:562. 86

20. Baguley D, McFerran D, Hall D. Tinnitus. Lancet Lond Engl. 2013;382(9904):1600–7.

21. Biswas R, Lugo A, Akeroyd MA, Schlee W, Gallus S, Hall DA. Tinnitus prevalence in Europe: a multi-country cross-sectional population study. Lancet Reg Health Eur. 2021;12:100250.

22. Clifford RE, Maihofer AX, Stein MB, Ryan AF, Nievergelt CM. Novel Risk Loci in Tinnitus and Causal Inference With Neuropsychiatric Disorders Among Adults of European Ancestry. JAMA Otolaryngol-- Head Neck Surg. 2020;146(11):1015–25.
