# Supplementary material for: Sex differences in the polygenic architecture of hearing problems in adults
Source: Genome Med. 2023 May 11;15:36. doi: 10.1186/s13073-023-01186-3 (PMC10173489; doi:10.1186/s13073-023-01186-3)
Supplement: Supplementary file 3 — Additional file 3: Figure S1. Manhattan plot of the sex-combined GWAS meta-analysis of UKB, NHS, and HPFS cohorts. The red line refers to the genome-wide significance threshold. Figure S2. Visual representation of the fine-mapping analysis for sex-combined meta-analysis. Each panel refers to a GWS risk locus. The variants were identified according to their LD with respect to the lead SNP and inclusion in the credible set with at most ten causal variants. Figure S3. Visual representation of the fine-mapping analysis for female meta-analysis. Each panel refers to a GWS risk locus. The variants were identified according to their LD with respect to the lead SNP and inclusion in the credible set with at most ten causal variants. Figure S4. Visual representation of the fine-mapping analysis for male meta-analysis. Each panel refers to a GWS risk locus. The variants were identified according to their LD with respect to the lead SNP and inclusion in the credible set with at most ten causal variants. [file 13073_2023_1186_MOESM3_ESM.docx]

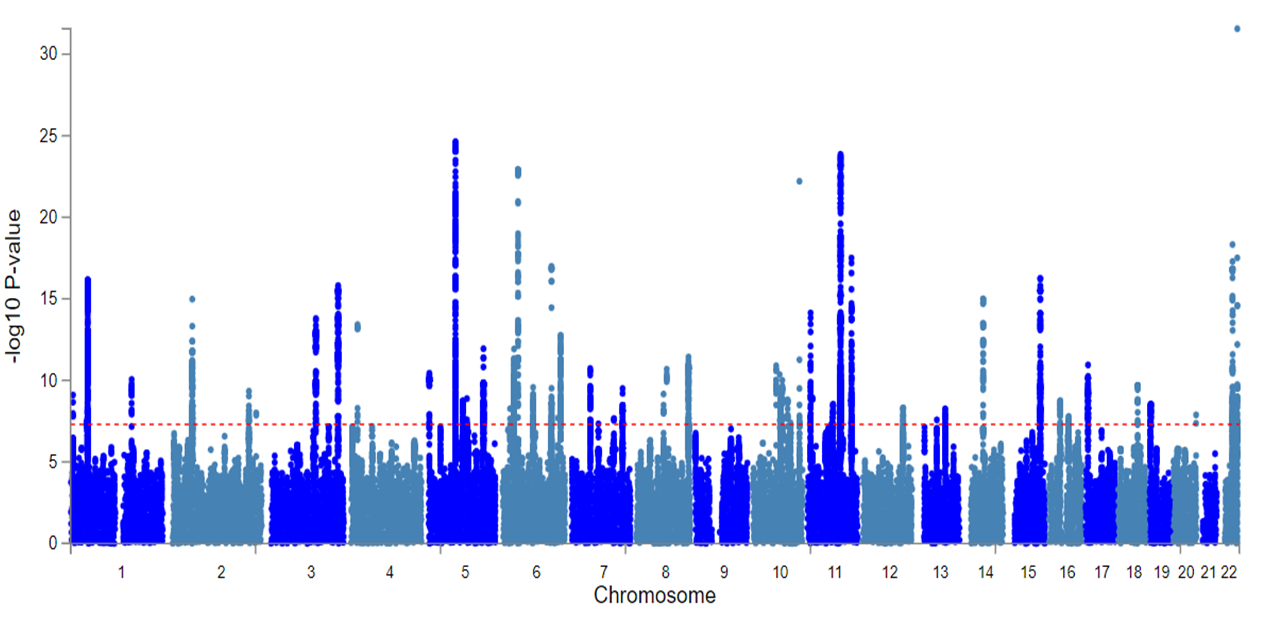


**Figure S1:** Manhattan plot of the sex-combined GWAS meta-analysis of UKB, NHS, and HPFS cohorts. The red line refers to the genome-wide significance threshold (p=5×10^-8^).


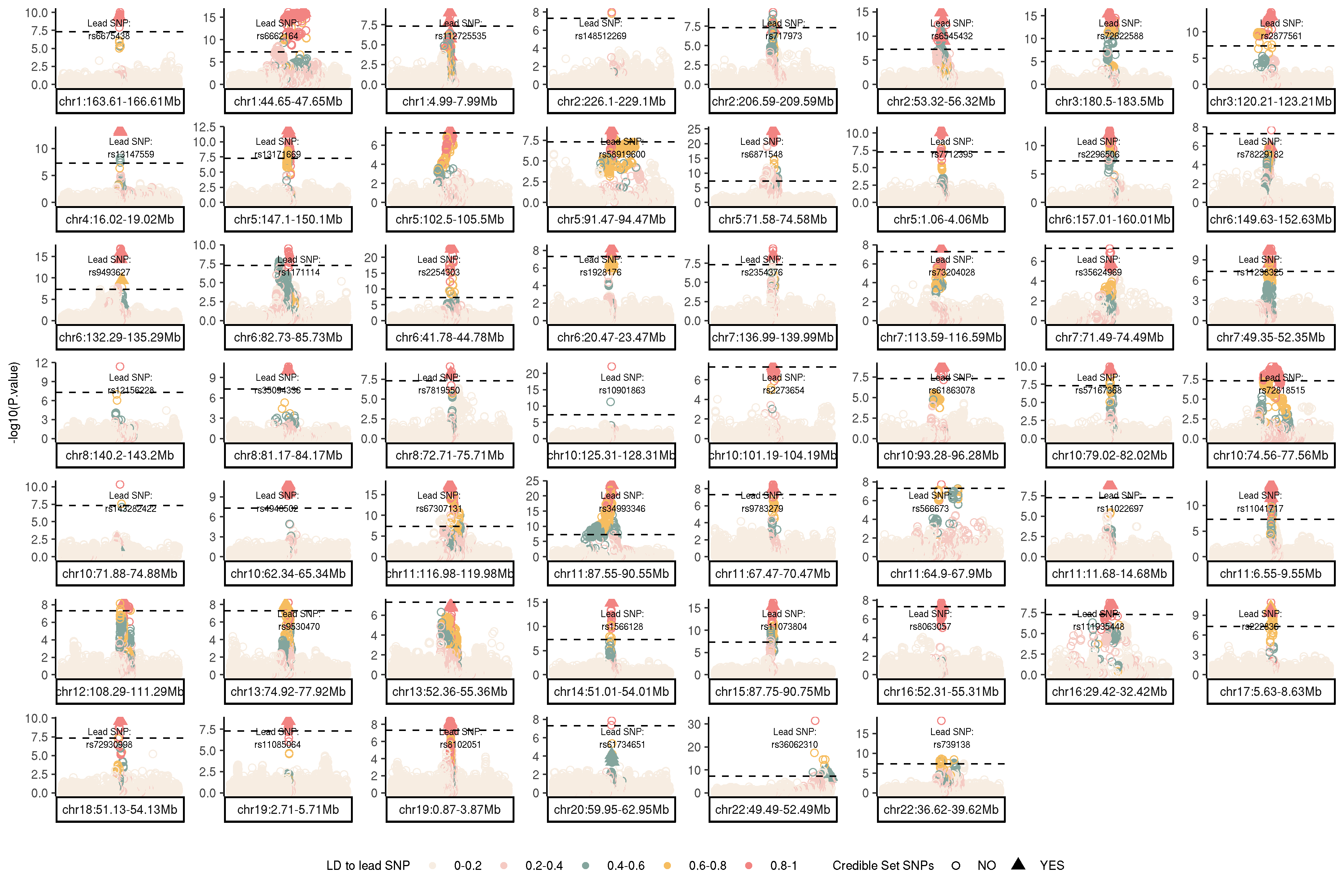


**Figure S2**: Visual representation of the fine-mapping analysis for sex-combined meta-analysis. Each panel refers to a GWS risk locus. The variants were identified according to their LD with respect to the lead SNP and inclusion in the credible set with at most ten causal variants.


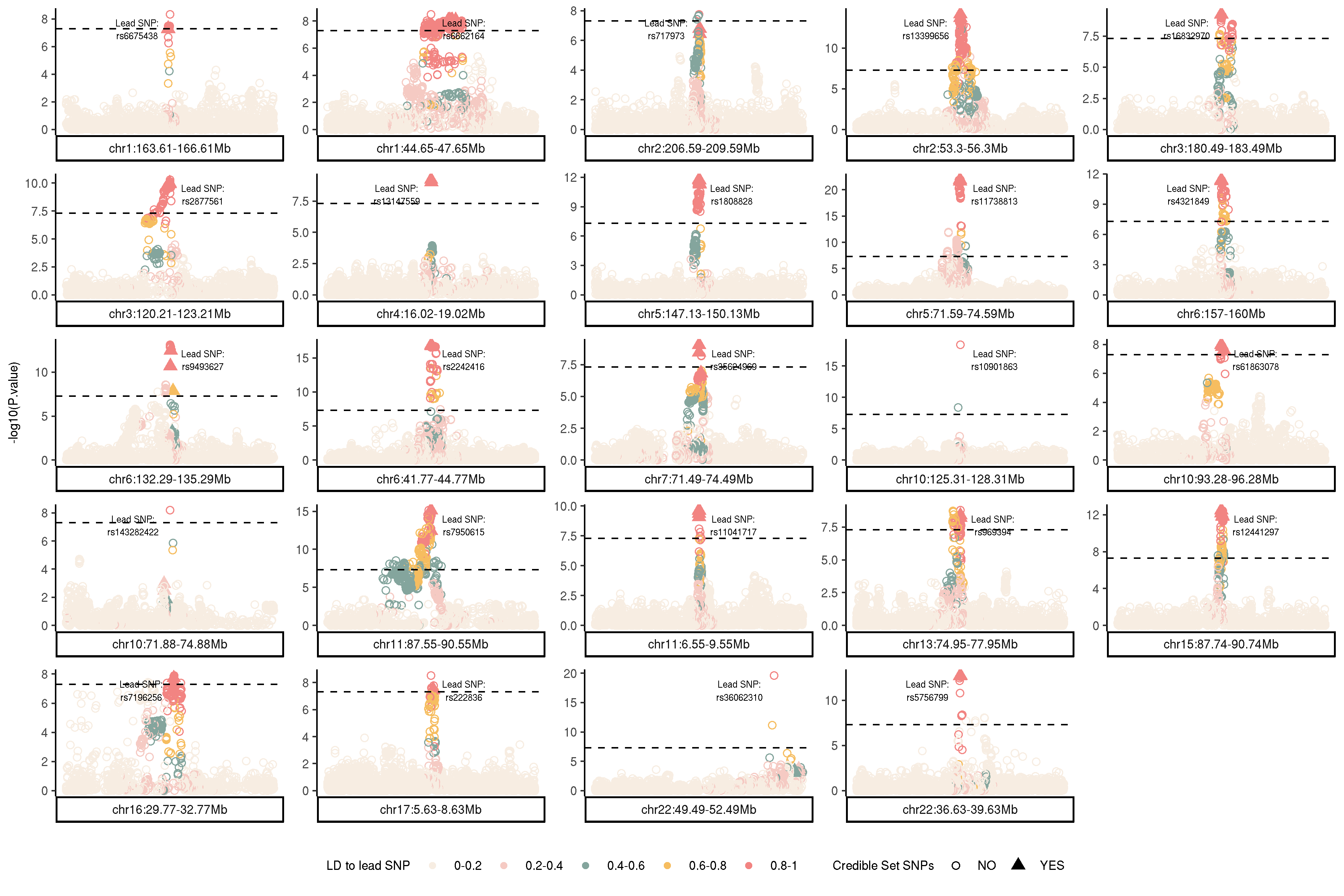


**Figure S3**: Visual representation of the fine-mapping analysis for female meta-analysis. Each panel refers to a GWS risk locus. The variants were identified according to their LD with respect to the lead SNP and inclusion in the credible set with at most ten causal variants.


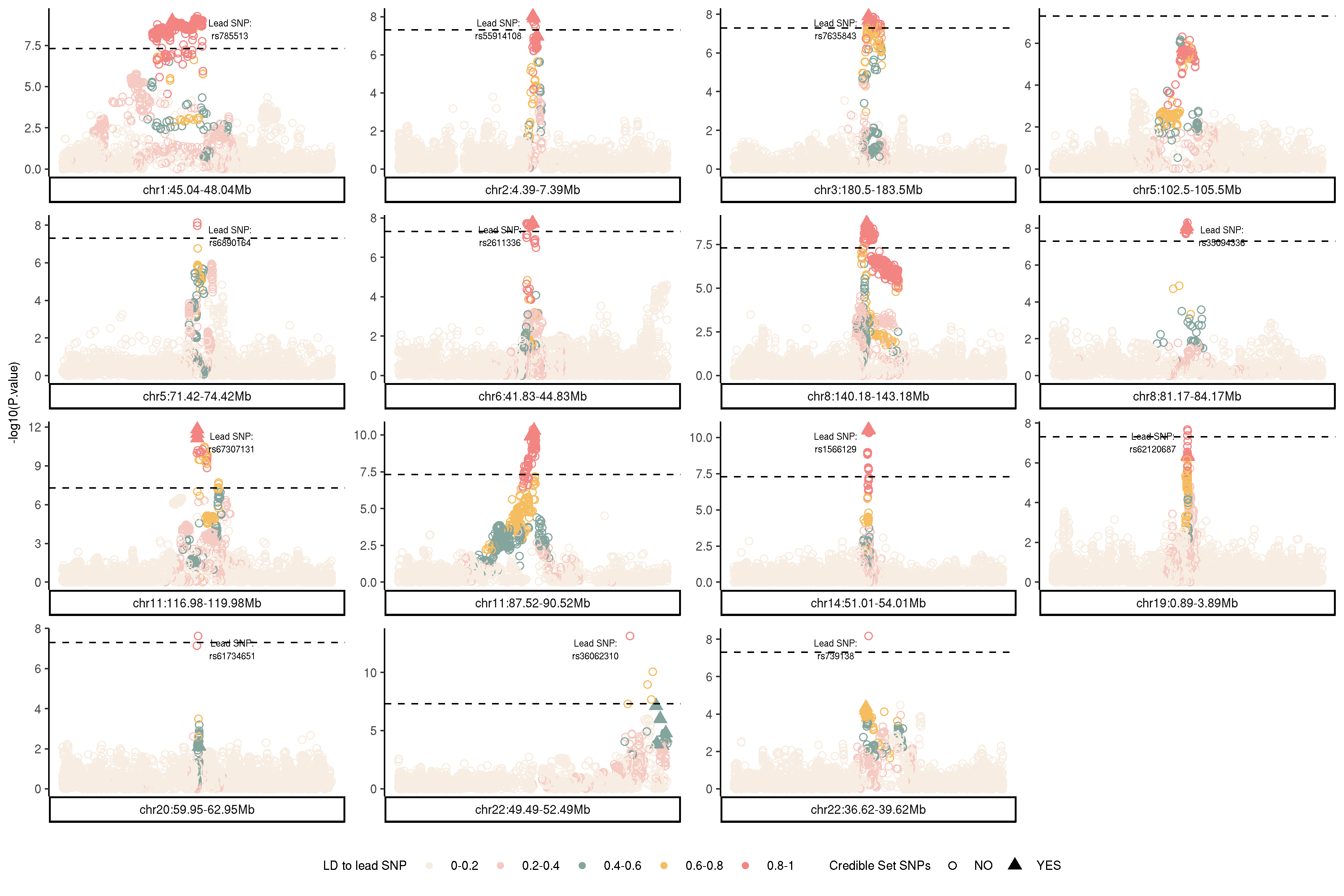


**Figure S4**: Visual representation of the fine-mapping analysis for male meta-analysis. Each panel refers to a GWS risk locus. The variants were identified according to their LD with respect to the lead SNP and inclusion in the credible set with at most ten causal variants.
